# PBR1 selectively controls biogenesis of photosynthetic complexes by modulating translation of the large chloroplast gene *Ycf1* in *Arabidopsis*

**DOI:** 10.1038/celldisc.2016.3

**Published:** 2016-05-10

**Authors:** Xiao-Fei Yang, Yu-Ting Wang, Si-Ting Chen, Ji-Kai Li, Hong-Tao Shen, Fang-Qing Guo

**Affiliations:** 1 The National Key Laboratory of Plant Molecular Genetics, Institute of Plant Physiology and Ecology, Shanghai Institutes for Biological Sciences, Chinese Academy of Sciences, Shanghai, China; 2 National Center of Plant Gene Research (Shanghai), Institute of Plant Physiology and Ecology, Shanghai Institutes for Biological Sciences, Chinese Academy of Sciences, Shanghai, China; 3 CAS Center for Excellence in Molecular Plant Sciences, Institute of Plant Physiology and Ecology, Shanghai Institutes for Biological Sciences, Chinese Academy of Sciences, Shanghai, China

**Keywords:** biogenesis, photosynthetic complex, RNA-binding protein, Ycf1

## Abstract

The biogenesis of photosystem I (PSI), cytochrome *b*_6_*f* (Cyt*b*_6_*f*) and NADH dehydrogenase (NDH) complexes relies on the spatially and temporally coordinated expression and translation of both nuclear and chloroplast genes. Here we report the identification of photosystem biogenesis regulator 1 (PBR1), a nuclear-encoded chloroplast RNA-binding protein that regulates the concerted biogenesis of NDH, PSI and Cyt*b*_6_*f* complexes. We identified *Ycf1*, one of the two largest chloroplast genome-encoded open reading frames as the direct downstream target protein of PBR1. Biochemical and molecular analyses reveal that PBR1 regulates *Ycf1* translation by directly binding to its mRNA. Surprisingly, we further demonstrate that relocation of the chloroplast gene *Ycf1* fused with a plastid-transit sequence to the nucleus bypasses the requirement of PBR1 for *Ycf1* translation, which sufficiently complements the defects in biogenesis of NDH, PSI and Cyt*b*_6_*f* complexes in *PBR1*-deficient plants. Remarkably, the nuclear-encoded PBR1 tightly controls the expression of the chloroplast gene *Ycf1* at the translational level, which is sufficient to sustain the coordinated biogenesis of NDH, PSI and Cyt*b*_6_*f* complexes as a whole. Our findings provide deep insights into better understanding of how a predominant nuclear-encoded factor can act as a migratory mediator and undergoes selective translational regulation of the target plastid gene in controlling biogenesis of photosynthetic complexes.

## Introduction

In green algae and vascular plants, the vast majority of plastid gene products are components either of photosynthetic enzymes or of the plastid gene expression machinery. Given that the thylakoid membrane complexes photosystem I (PSI), photosystem II (PSII), cytochrome *b*_6_*f* (Cyt*b*_*6*_*f*), ATP synthase and NADH dehydrogenase (NDH) each consist of both plastid and nuclear-encoded subunits, the stoichiometric accumulation of the various subunits of photosynthetic complexes in chloroplasts relies on a tight cooperation between the plastid and nucleus genomes [1, 2]. In photosynthetic eukaryotes, PSI reaction center complex is composed of 15 protein subunits (PsaA–PsaL and PsaN–PsaP) [3]. Among the 15 core subunits, 5 of these subunits (PsaA–PsaC, PsaI and PsaJ) are encoded in the chloroplast genome of higher plants, whereas the others are encoded by nuclear genes and posttranslationally imported into the chloroplast compartment [3, 4]. In contrast to PSII whose biogenesis has been studied in greatest detail [5, 6], the mechanisms for PSI biogenesis are still poorly understood. One reason for this is that a resolution of the different assembly intermediates is much more challenging than in the case of other photosynthetic complexes, presumably because the assembly process of PSI occurs very fast [7, 8]. A number of known factors are involved in the assembly of eukaryotic PSI, such as the hypothetical chloroplast reading frame 3 (Ycf3) [9, 10], a Ycf3-interacting protein Y3IP1 [11] and Ycf4 [12]. The majority of these assembly factors appear to be highly conserved between cyanobacteria, eukaryotic algae and higher plants [4, 7, 13].

Considering that PSI seems to accumulate to much higher levels than actually required to maintain electron flow, it is well established that linear electron flux between PSII and PSI is predominantly controlled at the level of the Cyt*b*_6_*f* complex [14, 15]. In addition, the Cyt*b*_6_*f* complex also functions in cyclic electron flow around PSI and in the generation of an electrochemical gradient across the thylakoid membrane used for ATP synthesis [16]. The Cyt*b*_6_*f* complex is composed of at least six chloroplast-encoded proteins (Cyt*f*, Cyt*b*_6_, PetD, PetG, PetL and PetN) and two nuclear-encoded proteins (PetC and PetM) [17]. Although the structure and function of the Cyt*b*_6_*f* complex of photosynthetic eukaryotes has been extensively investigated, less is known about its biogenesis in chloroplasts. In a variety of plant species, several nuclear-encoded factors have been reported to be essential for processing of specific Cyt*b*_6_*f* complex transcripts, including CRP1 in maize [18], MCA1 in *Chlamydomonas* [19] and PGR3, HCF152 and HCF153 in *Arabidopsis* [20–22].

Analysis of *Chlamydomonas* mutants has revealed that the photosynthetic membrane complexes underlie a protein assembly-dependent regulation of translation, the so-called control by epistasy of synthesis process, suggesting that translation seems to be the most important regulatory step during biogenesis of photosynthetic complexes [23, 24]. Clearly, the biogenesis of photosynthetic complexes requires the operation of highly efficient mechanisms for the coordinated expression and translation of both nuclear and chloroplast genes. Because of the complexity of this protein assembly-dependent regulation of translation, relatively little is known about regulatory mechanisms by which the nucleus tightly controls the expression of chloroplast genes via a set of *trans*-acting factors acting at the posttranscriptional level on a single (or a few) specific target mRNA(s) [1, 23].

In *Chlamydomonas*, PSI and Cyt*b*_6_*f* complexes form a protein supercomplex, which mediates cyclic electron transfer around PSI [25], whereas such a supercomplex has not yet been detected in land plants [26, 27]. Other than the structural components themselves, nuclear-encoded *trans*-acting factors involved in the concerted biogenesis of both PSI and Cyt*b*_6_*f* complexes remain to be identified. As a multi-subunit complex embedded in stroma thylakoids, the chloroplast NDH complex recycles electrons from ferredoxin (Fd) to plastoquinone (PQ) and subsequently to PSI through Cyt*b*_6_*f* complex in higher plants [28, 29]. On the basis of extensive mutant characterization, the NDH complex in flowering plants is postulated to be divided into four distinct subcomplexes: A, B, membrane-localized, and lumen-localized subcomplexes [30–32]. In higher plants, chlororespiratory reduction 1 (CRR1) is involved in biogenesis or stabilization of the NDH complex, possibly via the reduction of an unknown substrate [33]. Two Arabidopsis proteins, CRR6 and CRR7, identified in mutants specifically defective in NDH accumulation, are involved in posttranslational steps during the biogenesis of subcomplex A [34]. Because of its low abundance and fragility, little is known about the assembly process of the NDH complex.

Here we show that the nuclear-encoded S1-domain RNA-binding protein PBR1 represents a novel *trans*-acting factor that regulates the translation of *Ycf1*, one of the two largest chloroplast genome-encoded open reading frames [35], in the biogenesis of NDH, PSI and Cyt*b*_6_*f* complexes. Moreover, we provide compelling evidence that allotopic expression of the chloroplast gene *Ycf1* fused with a plastid-transit sequence in the nucleus can rescue the defects of NDH, PSI and Cyt*b*_6_*f* biogenesis in the *pbr1-1* mutant plants. We propose that the PBR1–Ycf1 module represents a novel mechanism for translational control of photosynthetic complex biogenesis in plants.

## Results

### PBR1 knockdown impairs biogenesis of NDH, PSI and Cytb_6_f Complexes

**Figure 1:**
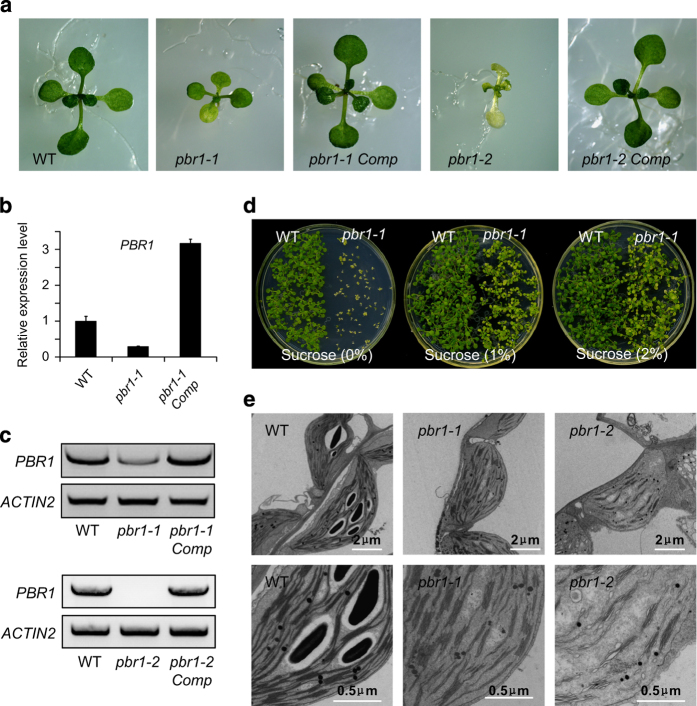
*PBR1* is required for chloroplast development. (**a**) Phenotypes of 12-day-old plants grown on ½ MS medium with 1% sucrose. (**b**, **c**) Expression levels of *PBR1* in the wild-type (WT), *pbr1-1*, *pbr1-2* and the complemented mutant plants of *pbr1-1* and *pbr1-2* analyzed by quantitative reverse transcriptase–PCR (RT–PCR) (**b**) and RT–PCR (**c**). *ACTIN2* was used as the internal standard. Error bars indicate s.d. of three technical replicates, and the results were consistent in three biological replicates. (**d**) Phenotypes of 10-day-old plants grown on ½ MS medium supplemented with different concentrations of sucrose. (**e**) Transmission electron micrographs of chloroplasts from the WT, *pbr1-1* and *pbr1-2*.

**Figure 2:**
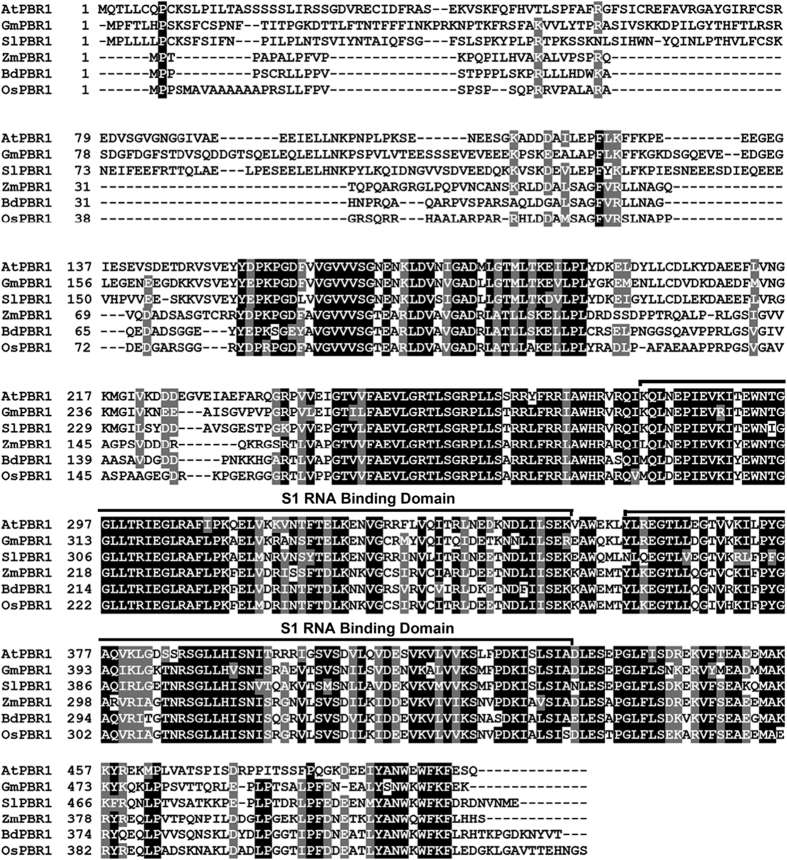
Alignments of derived amino-acid sequences of PBR1 homologs. Amino-acid sequences of AtPBR1 (At1g71720), GmPBR1 (GenBank accession number: XP_003535559), SlPBR1 (XP_004253265), ZmPBR1 (DAA58739), BdPBR1 (XP_003569284) and OsPBR1 (NP_001043440) were aligned as described in Materials and Methods. Identical amino-acid residues and conservative changes were depicted in black and grey background, respectively. Two S1 domains were labeled.

In contrast to extensive studies on pentatricopeptide repeat proteins [1, 36–39], little is known about the role of S1-domain RNA-binding proteins in posttranscriptional regulation of chloroplast-encoded mRNA particularly involved in the biogenesis of photosynthetic complexes. Toward functional characterization of S1-domain RNA-binding proteins in the chloroplast, the T-DNA insertion lines of *PBR1* were identified (Figure 1 and Supplementary Figure S1). Based on database searches and sequence alignments, *PBR1* is a single-copy gene in *Arabidopsis* and shares a significant identity with the homologous genes in different species of monocotyledonous and dicotyledonous plants (Figure 2 and Supplementary Figure S3). In addition, PBR1 consists of two S1 domains (Figure 2). The knockdown mutant plants of *pbr1-1* appeared slightly pale green with a reduced plant size (Figure 1a). The expression levels of *PBR1* in the *pbr1-1* and *pbr1-2* mutant seedlings were verified using quantitative PCR with reverse transcription (qRT–PCR) and RT–PCR (Figure 1b and c). Supplementation with sucrose in the medium significantly rescued the knockdown mutant *pbr1-1* (Figure 1d and Supplementary Figure S2). Unlike *pbr1-1*, the homozygous knockout mutant plants of *pbr1-2* were seedling-lethal when grown on sucrose-containing medium (Figure 1a). Furthermore, transforming genomic fragments of *PBR1* complemented the growth defects of both *pbr1-1* and *pbr1-2* mutants (Figure 1a), indicating that *PBR1* is the gene responsible for the pale-yellowish and growth-retarded phenotypes of the *pbr1* mutants.

**Figure 3:**
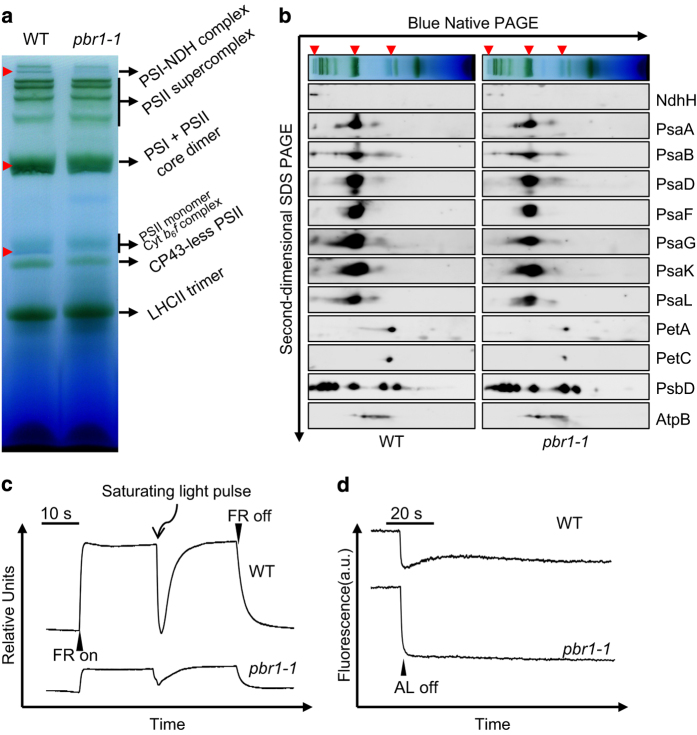
*PBR1* knockdown impairs biogenesis of NADH dehydrogenase (NDH), photosystem I (PSI) and cytochrome *b*_6_*f* (Cyt*b6f*) complexes. (**a**) Analysis of thylakoid membrane protein complexes by Blue-Native polyacrylamide gel electrophoresis (PAGE). Thylakoid membranes were isolated from wild-type (WT) and *pbr1-1* mutant leaves and equal amounts of samples (3 μg chlorophyll) were separated. Red arrows indicate the reduction of corresponding complexes in *pbr1-1* mutant. (**b**) Immunodetection of thylakoid complex proteins. The blots were probed with antibodies against the indicated proteins, respectively. (**c**) P700 redox kinetics determined by measuring absorbance of P700 at 820 nm induced by far-red light (FR, 720 nm). (**d**) NDH activities monitored by chlorophyll fluorescence. Leaves were exposed to actinic light (AL, 50 μmol of photons m^−2^ s^−1^) for 5 min. NDH activities were indicated as a transient increase in chlorophyll fluorescence after illumination. a.u., arbitrary units; SDS, sodium dodecyl sulfate.

Detailed examinations of ultrastructural alterations using transmission electron microscopy revealed that granum-stroma thylakoid membranes were disrupted in both the *pbr1-1* and *pbr1-2* mutants and the ultrastructural alterations were more pronounced in the knockout mutant *pbr1-2* (Figure 1e). Given the effect of knockdown or knockout of *PBR1* on ultrastructural alterations in chloroplast, especially for the severely disrupted stromal membranes in which PSI and Cyt*b*_6_*f* complexes are embedded, we next explored how *PBR1* influences the steady-state levels of photosynthetic complexes in thylakoid membranes. To this end, chlorophyll–protein complexes were separated by Blue-Native polyacrylamide gel electrophoresis (BN-PAGE). As shown in Figure 3a, the NDH–PSI supercomplex was detected as a high molecular weight green band in wild type [40], but only trace amount, if any, of the supercomplex could be detected in the *pbr1-1* mutant. There were two bands corresponding to PSII monomer and Cyt*b*_6_*f* complex in wild type, whereas the lower molecular band was almost completely missing in the mutant. Also, a considerable decrease was detected in the bands representing PSI and PSII core dimers in the *pbr1-1* mutant compared with that in wild type. By contrast, the amount of PSII supercomplex in *pbr1-1* remained largely unchanged in comparison to wild type (Figure 3a). These results suggest that *PBR1* knockdown is likely to selectively affect the biogenesis of NDH, PSI and Cyt*b*_6_*f* complexes.

Having established that PBR1 is involved in the biogenesis of NDH, PSI and Cyt*b*_6_*f* complexes, we next performed analyses of the two-dimensional sodium dodecyl sulfate PAGE (SDS-PAGE) gels in order to further examine the relative levels of the individual core subunits in these three complexes, respectively. Immunoblot analyses with antibodies against specific subunits showed that protein levels of the PSI and NDH subunits, including PsaA, PsaB, PsaD, PsaF, PsaL, PsaG, PsaK and NdhH, were nearly non-detectable in the band corresponding to the NDH–PSI supercomplex and substantially reduced in the bands representing PSI and PSII core dimers in the *pbr1-1* mutant compared with the subunit levels in wild type (Figure 3b and Supplementary Figure S4). In addition, the abundance of Cyt*b*_6_*f* core subunits (PetA and PetC) was also reduced in the *pbr1-1* mutant, whereas the abundance of PSII core subunit PsbD and ATP synthase complex subunit AtpB remained largely unchanged compared with wild type (Figure 3b and Supplementary Figure S4). Furthermore, the reduced abundance of the NDH, PSI and Cyt*b*_6_*f* subunits was validated in the *pbr1-1* and *pbr1-2* mutants, respectively, by immunoblotting alongside with a dilution series of a wild-type sample (Supplementary Figure S5). These results further support the hypothesis that PBR1 has a critical role in the regulation of biogenesis of NDH, PSI and Cyt*b*_6_*f* complexes.

### Photosynthetic electron flow is impaired in pbr1-1

To further characterize the effect of *PBR1* knockdown-induced reduction in the abundance of NDH, PSI and Cyt*b*_6_*f* complexes on photosynthetic electron flow, we conducted measurements for the light responses of P700 redox state and PSI–NDH activity, respectively. The reduction level in PSI reaction center was monitored by absorbance changes at 820 nm and the maximum oxidation level of P700 was determined by far-red light illumination [21, 41]. As shown in Figure 3c, under far-red light background, the maximum oxidation level of P700 was severely reduced, and the reduction level of P700 triggered by a saturating flash light was dramatically lower in the *pbr1-1* mutant compared with the oxidation and reduction patterns of P700 in wild type. Thus the dramatic decrease in the maximum oxidation level of P700 in *pbr1-1* represents the capacity limitation in electron acceptance from PSI, which is in agreement with the reduced abundance of PSI caused by *PBR1* mutations.

Consistent with the results of BN-PAGE and immunoblot analyses that the NDH–PSI supercomplex was almost completely missing in *pbr1-1* (Figure 3a and b), the mutant showed no transient increase in fluorescence after the actinic light (AL) was switched off (Figure 3d), indicating that NDH activity was severely impaired. We conclude that *PBR1* is essential for NDH activity.

### PBR1 knockdown inhibits induction of non-photochemical quenching (NPQ)

**Figure 4:**
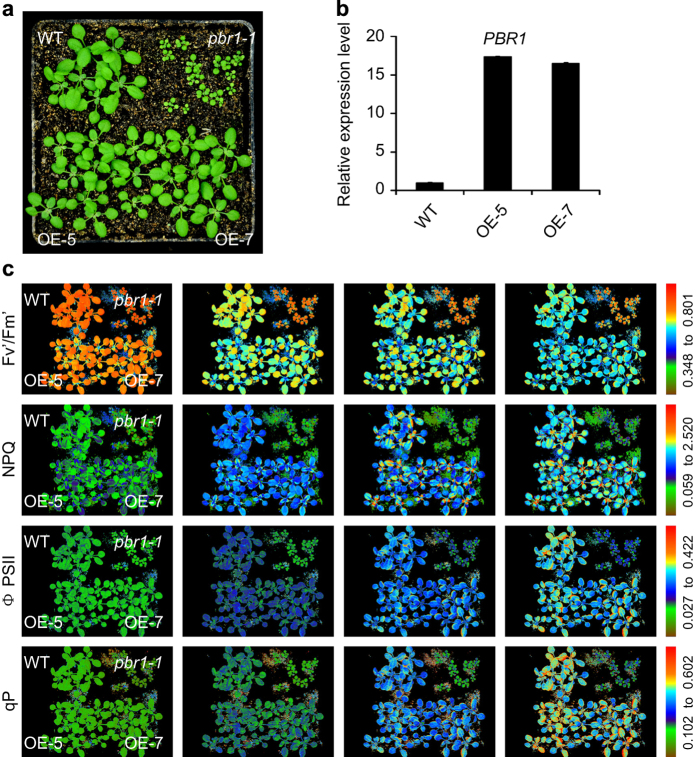
Photosynthetic electron flow is disrupted in *pbr1-1.* (**a**) Phenotypes of 16-day-old plants of the wild-type (WT), *pbr1-1* and *PBR1* overexpression lines (OE-5 and OE-7) grown in greenhouse. (**b**) Expression levels of *PBR1* in the WT and *PBR1* overexpression lines (OE-5 and OE-7) analyzed by quantitative reverse transcriptase–PCR. (**c**) Measurements of photosynthetic parameters in leaves of the indicated genotypes as shown in **a**. The plants were exposed to very bright saturating pulses of light (flashing once every 30 s in 5 min), and fluorescence images were shown at the first, second, fifth and tenth flash (from left to right). Fluorescence was measured with CF Imager and visualized using a pseudocolor index, as indicated on the right.

In order to test the effect of *PBR1* accumulation on NPQ processes, the transgenic lines overexpressing *PBR1* were generated (Figure 4a and b). In Figure 4c, the representative induction patterns of NPQ were shown for the wild type, *pbr1-1* and two overexpression lines of *PBR1* (OE-5 and OE-7) at four flash points. As expected, NPQ was not able to be significantly induced for the *pbr1-1* mutant by the multiple flashes of a brief very bright saturating light, whereas in the wild type, NPQ was rapidly induced and gradually increased under the same induction conditions. It was noted that no significant difference was observed in NPQ induction patterns between the wild type and two overexpression lines (Figure 4c, NPQ image panels; and Supplementary Figure S6B). Generally speaking, the changes in Fv′/Fm′ can be used as an indicator of changes in NPQ as the two parameters will coincide [42]. Consistent with damage in photosynthetic electron flow caused by the reduced accumulation of PSI and Cyt*b*_6_*f* complexes (Figure 3a and b), the *pbr1-1* mutant showed a high chlorophyll fluorescence (*hcf*) phenotype during the flashes, indicating that absorbed light energy cannot be used for photosynthesis and is instead emitted as red fluorescent light in the mutant plants (Figure 4c, Fv′/Fm′ image panels; and Supplementary Figure S6A). In contrast to the mutant, the values of Fv′/Fm′ were rapidly reduced in both the wild type and two overexpression lines. Because of an impairment in the electron transport capacity, the operating efficiency of PSII photochemistry (ϕPSII) in the *pbr1-1* mutant was consistently lower compared with the increased levels of ϕPSII in the wild type and two overexpression lines during the flashes (Figure 4c, ϕPSII image panels; and Supplementary Figure S6C). As shown in Figure 4c (qP image panels and Supplementary Figure S6D), the levels of qP in the *pbr1-1* mutant remained largely unchanged during the multiple flashes, indicating that a greater proportion of reaction centers in PSII could be closed, which will inevitably cause a decline in quantum efficiency of PSII in the mutant as represented in ϕPSII image panels in Figure 4c. Taken together with the immunological data and spectroscopic analyses for the light responses of P700 redox state and PSI–NDH activity (Figure 3), these results described above suggest that *PBR1* knockdown impairs energy flow via the linear electron transport chain and cyclic electron transport around PSI and therefore perturbs induction of thermal dissipation owing to the lack of PSI–NDH supercomplex, an essential component for acidifying the thylakoid lumen that triggers NPQ induction processes.

### PBR1 modulates translation of Ycf1 by directly binding to its mRNA

**Figure 5:**
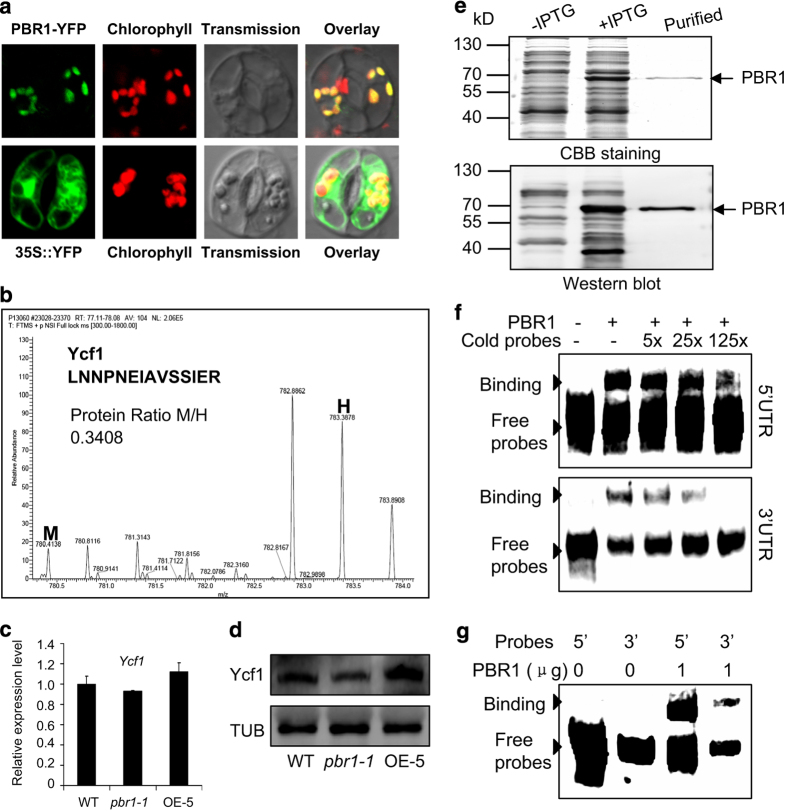
PBR1 regulates *Ycf1* translation by binding to its mRNA. (**a**) PBR1-YFP (yellow fluorescent protein) signals are targeted to chloroplasts as examined in guard cells of epidermal peels. (**b**) The representative mass spectra for identification of Ycf1 protein in samples extracted from wild-type (WT) and *pbr1-1* mutant leaves pulse-labeled with ‘heavy’ (H) and ‘medium heavy’ (M) stable isotope amino acids, respectively. The ratio of peak intensities of M versus H peptides reflects difference between the *pbr1-1* mutant and WT in translation of the corresponding proteins as the newly synthesized proteins incorporate either the M or H amino acids. (**c**, **d**) The expression and protein levels of Ycf1 in WT, *pbr1-1* and *PBR1* overexpression line OE-5 analyzed by quantitative reverse transcriptase–PCR (**c**) and western blots (**d**), respectively. An Ycf1 polyclonal antibody was used, and equal protein loading was confirmed with antiserum against α-Tubulin (TUB). (**e**) Expression and purification of recombinant PBR1 protein in *E. coli.* (**f**, **g**) RNA-binding activity of PBR1 was detected by electrophoretic mobility shift assay (EMSA). Proteins were incubated with biotin-labeled probes derived from the 5′-UTR (untranslated region) or 3′-UTR of *Ycf1* mRNA and separated by 6% native polyacrylamide gel electrophoresis. The specificity of binding bands was confirmed by cold probe competition.

As predicted, PBR1 is targeted to chloroplasts (Figure 5a). Based on sequence alignments, the central and C-terminal parts of PBR1 form an elongated RNA-binding domain comprising two repetitions of a conserved structural domain called S1-motif (Figure 2). According to existing literatures, S1-motif interacts with mRNA during translation initiation [36, 43, 44]. Thus earlier studies on S1-motif led us to reason that PBR1 might act as a nuclear-encoded *trans*-acting factor in the regulation of biogenesis of NDH, PSI and Cyt*b*_6_*f* complexes at the translational level. We proposed a model in which PBR1 may regulate the translation of a key mediator protein encoded by a chloroplast gene that might function in the biogenesis of NDH, PSI and Cyt*b*_6_*f* complexes.

**Figure 6:**
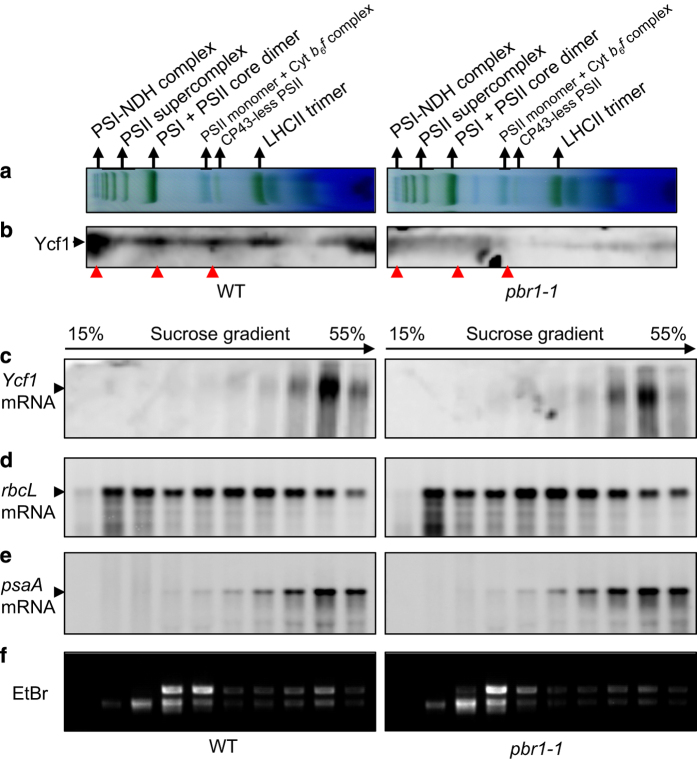
*PBR1* knockdown impairs accumulation of the Ycf1 protein in thylakoid membranes and association of the *Ycf1* transcript with polysomes. (**a**) Analysis of thylakoid membrane protein complexes by Blue-Native polyacrylamide gel electrophoresis. Thylakoid membranes were isolated from wild-type (WT) and *pbr1-1* mutant leaves and equal amounts of samples (3 μg chlorophyll) were separated. (**b**) The protein levels of Ycf1 analyzed by western blots in thylakoid membranes of the WT and *pbr1-1* with an polyclonal antibody against Ycf1. Red arrows indicate the reduction in the protein levels of Ycf1 in the corresponding complexes in the *pbr1-1* mutant compared with WT. (**c**–**e**) Association of the *Ycf1*, *rbcL* and *psaA* transcripts with polysomes. Whole-cell extracts were fractionated in linear 15–55% sucrose gradients by ultracentrifugation. Ten fractions with equal volume were collected from top to bottom of the sucrose gradients, and equal proportions of each fraction were analyzed by northern blotting with specific probes of *Ycf1* (**c**), *rbcL* (**d**) and *psaA* (**e**), respectively. (**f**) RNA gel blots of polysome gradient fractions were stained with ethidium bromide (EtBr) to visualize distribution of rRNAs.

To test this hypothesis, we examined the differences in translation efficiency of thylakoid membrane proteins encoded by chloroplast DNA in the band corresponding to the NDH–PSI supercomplex, as shown in Figure 3a, between the *pbr1-1* and wild type by employing the pulsed stable isotope labeling assay with amino acids [45]. In addition, the comparative analysis between wild type and OE-5, one of the *PBR1*-overexpression lines, was also conducted with gain-of-function studies. Thylakoid membranes were extracted from wild-type and *pbr1-1* mutant or OE-5 leaves incorporated with heavy isotope-labeled amino acids (H) and medium heavy isotope-labeled amino acids (M), respectively and the NDH–PSI supercomplex was separated by BN-PAGE. The proteins in the corresponding band excised from BN-PAGE gel were digested with trypsin and identified by matrix-assisted laser desorpton/ionization time-of-flight/time-of-flight mass spectra analysis. As shown in Figure 5b, the translation efficiency of Ycf1 in chloroplast was dramatically reduced by 66% in the *pbr1-1* mutant leaves compared with that in wild-type leaves. By contrast, overexpressing *PBR1* significantly increased the translation efficiency of Ycf1 by 54% in the leaves of overexpression line OE-5 compared with that in wild-type leaves (Supplementary Figure S7). Indeed, Ycf1 was the only candidate protein whose translation efficiency was downregulated in the *pbr1* mutant plants and upregulated in the *PBR1* overexpression plants, which is the major reason why we chose Ycf1 as the most possible candidate rather than other proteins (Supplementary Table S1). Furthermore, the protein levels of Ycf1 were in agreement with the translation efficiency detected in the leaves of three genotypes mentioned above when validated using western blots with a polyclonal antibody against Ycf1 (Figure 5d). Also, a considerable loss in abundance of Ycf1 was detected in the bands representing the NDH–PSI supercomplex, PSI and PSII core dimers and Cyt*b*_6_*f* complex in the *pbr1-1* mutant compared with that in wild type when western blots were performed with thylakoid membrane protein complexes analyzed by BN-PAGE (Figure 6a and b). It should be noted that the transcript levels of *Ycf1* remained largely unchanged among wild type, *pbr1-1* and overexpression line OE-5 (Figure 5c). Taken together, these results demonstrate that PBR1 regulates the biosynthesis of Ycf1 that may be the possible mediator protein involving biogenesis of NDH, PSI and Cyt*b*_6_*f* complexes.

It is well known that RNA–protein interactions govern the action of the nuclear-encoded *trans*-factors that act most often on the 5′-UTR (untranslated region) of their organelle mRNA targets [1, 23]. To determine whether PBR1 directly binds to the UTR regions of the *Ycf1* mRNA, we performed eletrophoretic mobility shift assay (EMSA) with the purified recombinant PBR1 proteins (Figure 5e). Direct interaction of the purified PBR1 proteins with the 5′- or 3′-UTR of *Ycf1* mRNA is shown in Figure 5f and g. It is noteworthy that the binding affinity of PBR1 for the 5′-UTR of *Ycf1* mRNA was found to be higher than the affinity for the 3′-UTR (Figure 5f and g). The specificity of these binding bands was confirmed by cold oligonucleotide competition (Figure 5f).

Interestingly, a previous report showed that the protein level of Rubisco large subunit was dramatically reduced in the RLSB (PBR1)-silenced plants, suggesting that knockdown of *RLSB* (*PBR1*) affects accumulation of *rbcL* mRNA as well as synthesis of large subunit protein [46]. Unfortunately, we cannot detect any difference in the abundance of large subunit protein between *pbr1-1* mutant and wild-type plants grown on peat soil for 16 days (Supplementary Figure S8). On the other hand, we performed competition experiments using the 5′-UTR of *rbcL* mRNA as a cold oligonucleotide competitor to further test the binding specificity of PBR1 to the 5′-UTR of *Ycf1*. As shown in Supplementary Figure S9B, adding the cold probe 5′-UTR of *rbcL* mRNA failed to compete with the hot probe 5′-UTR of *Ycf1* mRNA, even in 250-fold higher concentration. Bowman *et al*. [46] have shown that RLSB (PBR1) binds with high affinity to 5′-UTR of the *rbcL* mRNA [46]. We performed RNA-binding assay to test the binding activity of PBR1 to 5′-UTR of the *rbcL* mRNA. As shown in Supplementary Figure S9C, strong binding signals were detected, indicating that RLSB (PBR1) can bind to the 5′-UTR of *rbcL* mRNA with high affinity, which is consistent with the results reported by Bowman *et al*. [46]. However, the binding signals were not reduced in the presence of increasing amounts of unlabeled 5′-UTR of the *rbcL* mRNA, even in 125-fold higher concentration (Supplementary Figure S9C). Similar results were obtained when 5′-UTR of the *rbcL* mRNA was used as a cold oligonucleotide competitor to compete with the labeled 5′-UTR of the *Ycf1* mRNA in competition experiments (Supplementary Figure S9B). These results indicate that RLSB (PBR1) binds to 5′-UTR of the *rbcL* mRNA, but this kind of binding is not specific because adding the ‘cold’ probes of the *rbcL* 5′-UTR, even in 125- or 250-fold higher concentration, failed to compete with the ‘hot’ probes of the labeled *rbcL* 5′-UTR or the *Ycf1* 5′-UTR. In addition, we have also examined the binding activity of PBR1 to 5′-UTR of the *psaA* mRNA and found no detectable binding signals (Supplementary Figure S9D). Moreover, the binding activity of PBR1 to 5′-UTR of the *Ycf1* mRNA remained largely unchanged with high concentration of NaCl treatment (150 mM) (Supplementary Figure S9A). Thus the binding specificity of PBR1 to 5′-UTR of the *Ycf1* mRNA has been further confirmed.

To further confirm that the reduced accumulation of Ycf1 in the *pbr1-1* mutant is caused by a defect in translation, we performed experiments to examine whether there is a difference in polysomal association of the *Ycf1* mRNA between wild-type and *pbr1-1* mutant. The results showed that polysomal association of the *Ycf1* mRNA was substantially reduced in the *pbr1-1* mutant compared with that in wild type when examined by northern blotting (Figure 6c and Supplementary Figure S10). Based on the images of EtBr staining, the total RNA loading between wild type and *pbr1-1* was very similar (Figure 6f). However, no significant difference between wild-type and *pbr1-1* mutant was detected in polysomal association of the *rbcL* mRNA or *psaA* mRNA, which can be taken as the reference mRNAs (Figure 6d and e). These results imply that the reduced accumulation of the Ycf1 protein in *pbr1-1* is mainly the result of impaired translation. These results, combined with translation efficiency data, strongly suggest that PBR1 modulates translation of the *Ycf1* mRNA by binding to its UTR regions.

### Allotopic expression of Ycf1 in the nucleus restores biogenesis of NDH, PSI and Cytb_6_f complexes in PBR1-deficient plants

**Figure 7:**
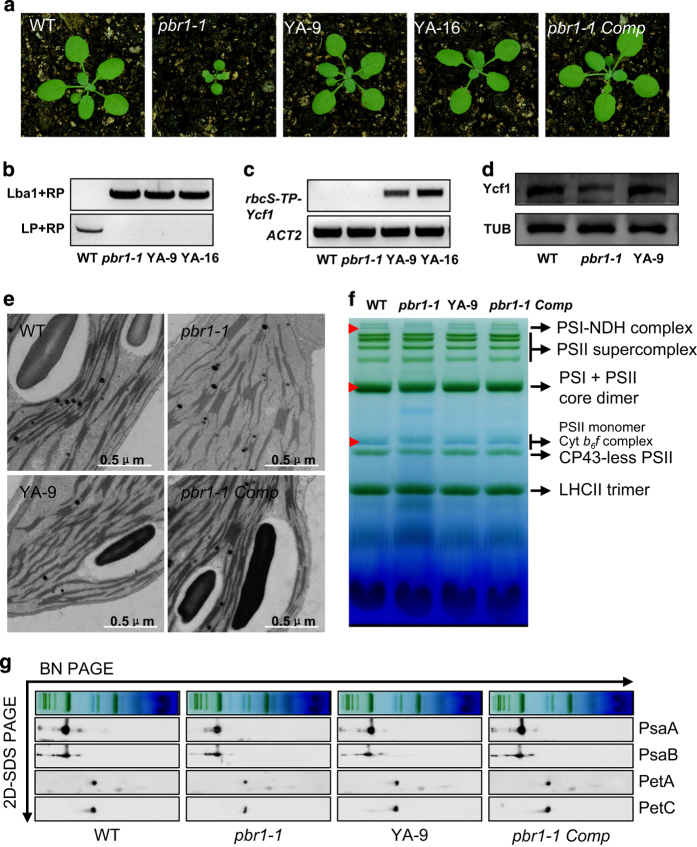
Relocation of *Ycf1* in nucleus restores biogenesis of NADH dehydrogenase (NDH), photosystem I (PSI) and cytochrome *b*_6_*f* (Cyt*b*_6_*f*) in *pbr1-1*. (**a**) Phenotypes of 16-day-old plants of the wild-type (WT), *pbr1-1*, two allotopic-expression lines of *Ycf1* (YA-9 and YA-16) and complemented *pbr1-1* with genomic DNA of *PBR1* (*pbr1-1 Comp*). (**b**, **c**) Allotopic expression analysis of *Ycf1* fused with the plastid-transit peptide sequence of the nuclear-encoded *rbcS* gene (*rbcS-TP-Ycf1*) by reverse transcriptase–PCR in leaves of the indicated genotypes. The specific primers derived from the plastid-transit sequence of *rbcS* and coding region of *Ycf1* were used to amplify the transcripts of *rbcS-TP-Ycf1* from the nucleus. (**d**) Protein levels of Ycf1 in leaves of the WT, *pbr1-1* and YA-9 plants detected by western blots. (**e**) Transmission electron micrographs of chloroplasts from the indicated genotypes. (**f**) Analysis of thylakoid membrane protein complexes by Blue-Native polyacrylamide gel electrophoresis (BN-PAGE). Equal amounts of samples (3 μg chlorophyll) were separated. Red arrows indicate the restoration of corresponding complexes in *pbr1-1* mutant by allotopic expression of *Ycf1* or genomic complementation. (**g**) Immunodetection of thylakoid complex proteins. The blots were probed with antibodies against the indicated proteins, respectively. SDS-PAGE, sodium dodecyl sulfate PAGE.

Next we investigated whether the requirement of PBR1 for biogenesis of NDH, PSI and Cyt*b*_6_*f* complexes is due to defects in *Ycf1* translation caused by *PBR1* knockdown. Given that PBR1 is required for *Ycf1* translation in chloroplasts, relocation of the chloroplast gene *Ycf1* fused with a plastid-transit sequence to the nucleus will bypass the requirement of PBR1 for *Ycf1* translation in chloroplast. Toward this end, we generated the transgenic lines of the *pbr1-1* mutant plants expressing the chloroplast gene *Ycf1* fused with the plastid-transit sequence of the nuclear-encoded *rbcS* gene, encoding the small subunit of Rubisco. Strikingly, allotopic expression of *Ycf1* in the nucleus rescued the growth-retarded phenotypes of the *pbr1-1* mutant plants to the growth levels of the wild-type and complemented *pbr1-1* mutant plants transformed with genomic fragments of *PBR1* (Figure 7a–c). More importantly, the increased protein levels of Ycf1 in the allotopic expression line *of Ycf1* (YA-9) were validated by western blots (Figure 7d). In agreement with restoration in growth, the allotopic expression of *Ycf1* in the nucleus complemented defects in biogenesis of NDH, PSI and Cyt*b*_6_*f* complexes in the *pbr1-1* mutant plants (Figure 7f and g). Furthermore, the complementation with the nuclear *rbcS-TP-Ycf1* gene sufficiently rescued the ultrastructural disruption of granum-stroma thylakoid membranes in the chloroplasts of the *pbr1-1* mutant (Figure 7e). Collectively, several lines of evidence described above suggest that the ability of PBR1 to exert its physiological regulatory function on biogenesis of NDH, PSI and Cyt*b*_6_*f* complexes depends on *Ycf1* translation.

Based on bioinformatics analysis, Ycf1 is a predicted coiled-coil (CC) protein containing two CC domains, designated as CC1 and CC2 (Supplementary Figure S11A). Given that CCs are well-characterized protein–protein interaction domains [47], we tested the interaction possibility of CC domains (CC1 and CC2) of the Ycf1 protein with the representative subunits of PSI and Cyt*b*_6_*f* complexes and ATP synthase complexes in a split-ubiquitin yeast two-hybrid (SUY2H) system. The results showed that the CC1 domain strongly interacted with PsaA and PsaB but weakly with PetA (Supplementary Figure S11B). A weaker interaction of the CC2 domain with PsaA, PsaB and PetA was also observed (Supplementary Figure S11B). In contrast, no interaction was detected between the CC domains (CC1 and CC2) and the representative subunit AtpB of the ATP synthase complex (Supplementary Figure S11B). These results indicate that Ycf1 could selectively interact with the PSI and Cyt*b*_6_*f* complexes.

As a 22-kD protein with two transmembrane domains, Ycf4 is localized in thylakoid membranes [10]. This protein is encoded by the chloroplast genome and is highly conserved in photosynthetic organisms from cyanobacteria to higher plants [10]. Because Ycf1 was identified in a stable Ycf4-containing complex essential for the accumulation of PSI in *Chlamydomonas reinhardtii* [12], we have also examined whether the CC domains of Ycf1 could interact with Ycf4 using the same SUY2H system as mentioned above. We found that the CC domains of Ycf1 strongly interact with Ycf4 (Supplementary Figure S11B).

### Accumulation of PBR1 and Ycf1 accommodates the concerted biogenesis of photosynthetic complexes during chloroplast development

**Figure 8:**
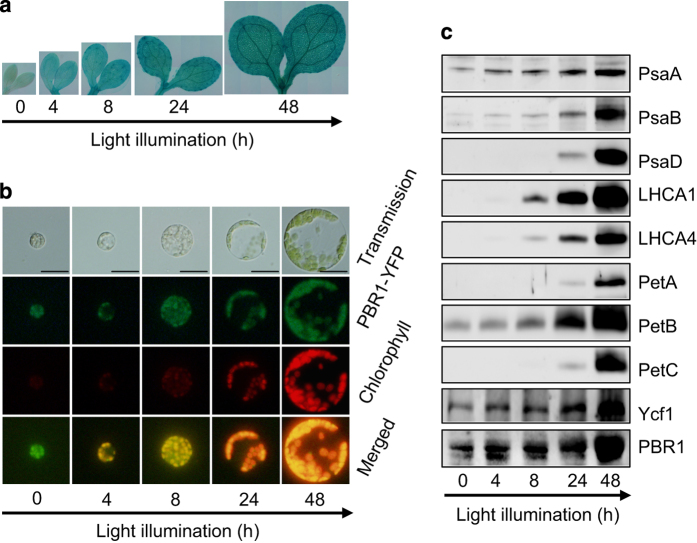
Accumulation of PBR1 and Ycf1 is light-inducible during chloroplast development. (**a**) GUS-staining patterns of representative etiolated seedlings harboring *Pro-PBR1::GUS* constructs when exposed to light in 48 h. (**b**) Subcellular localization of PBR1-YFP (yellow fluorescent protein) during chloroplast maturation in protoplasts isolated from the cotyledons of representative etiolated seedlings harboring *35S::PBR1-YFP* constructs during light illumination in 48 h. Bars=20 μm. (**c**) Immunodetection of accumulation of PBR1 and Ycf1 and abundance of thylakoid membrane proteins. The blots were probed with antibodies against the indicated proteins, respectively. Proteins were extracted from the wild-type etiolated seedlings during light illumination in 48 h. Protein samples were loaded on equal amounts of seedling fresh weight.

To test whether PBR1 is a light-inducible gene, GUS activities were examined in the *ProPBR1::GUS* transgenic plants. The results showed that *PBR1* was highly expressed in greening of the transgenic etiolated seedlings in a light-inducible way (Figure 8a). Interestingly, PBR1 was targeted to etioplasts in protoplasts prepared from cotyledons of the transgenic etiolated seedlings harboring *35S::PBR1-YFP* (yellow fluorescent protein) constructs (Figure 8b). We next examined the protein levels of PBR1 and Ycf1 as well as the accumulation of subunits of PSI and Cyt*b*_6_*f* during light-induced greening. The western blots indicated that PBR1 and Ycf1 accumulated even in etiolated seedlings and gradually increased during 48-h exposure to light (Figure 8c). Our data indicate that the levels of PBR1 and Ycf1 proteins are upregulated to accommodate the high demand for the concerted biogenesis of both PSI and Cyt*b*_6_*f* during chloroplast development.

## Discussion

In this study, we report that PBR1, a nuclear-encoded S1-domain containing RNA-binding protein, functions as a translational activator of *Ycf1*, serving to control biogenesis of NDH, PSI and Cyt*b*_6_*f* complexes. Furthermore, several lines of evidence support that PBR1 has a direct role in activating *Ycf1* translation by binding to the UTR regions of *Ycf1* mRNA, which fits with the notion that PBR1 functions as a S1-domain containing RNA-binding protein. Finally, we show compelling evidence that relocation of the chloroplast gene *Ycf1* fused with a plastid-transit sequence to the nucleus bypasses the requirement of PBR1 for *Ycf1* translation, which sufficiently complements defects in the biogenesis of NDH, PSI and Cyt*b*_6_*f* complexes in *PBR1*-deficient plants. It truly surprised us to find that Ycf1 from its cytoplasmic berth can function physiologically in the concerted biogenesis of NDH, PSI and Cyt*b*_6_*f* complexes. Together, these findings reveal a new regulatory paradigm in which PBR1 serves as a translational activator of *Ycf1* that is an essential component in controlling the selective biogenesis of photosynthetic complexes in *Arabidopsis*.

A prominent feature of PBR1 is the presence of two repetitions of a conserved structural domain called S1 domain. This structural motif can bind single-strand RNA with some sequence specificity and is found in a diversity of proteins functioning in RNA metabolism in all organisms [48–50]. PBR1 shares a number of characteristics that are compatible with a role as a translational activator. First, PBR1 likely modulates the translation efficiency of *Ycf1* in chloroplast, thereby controlling the concerted biogenesis of NDH, PSI and Cyt*b*_6_*f* complexes (Figures 5 and 6). In accordance with this, knockdown of *PBR1* mRNA levels severely suppressed photosynthetic electron flow caused by the reduced accumulation of NDH, PSI and Cyt*b*_6_*f* complexes (Figures 3 and 4 and Supplementary Figure S6). Second, PBR1 interacts with the *Ycf1* mRNA by directly binding to its 5′-UTR with a relatively higher affinity rather than to its 3′-UTR (Figure 5), which is in agreement with most of the case studies documented so far in which the nuclear factors bind to the 5′-UTR of the chloroplast transcripts [1, 23, 24]. Third, our results with the allotopic expression of *Ycf1* in the nucleus illustrate how PBR1 controls biogenesis of NDH, PSI and Cyt*b*_6_*f* complexes through modulation of *Ycf1* translation (Figure 7). Our findings thus highlight the importance of dissecting the PBR1–Ycf1 module for translational control of the concerted biogenesis of NDH, PSI and Cyt*b*_6_*f* complexes.

It should be noted that our findings are not consistent with a previous report showing that knockdown of *RLSB* (*PBR1*) affects accumulation of *rbcL* mRNA as well as synthesis of RbcL protein [46]. Based on several lines of evidence presented in this study, it is unlikely that *RbcL* is a downstream target of *RLSB* (*PBR1*) in *Arabidopsis* because (i) we cannot detect any difference in the abundance of RbcL protein between wild-type and *pbr1-1* mutant (Supplementary Figure S8), (ii) no significant difference was detected in polysomal association of the *rbcL* mRNA between wild-type and *pbr1-1* mutant (Figure 6d), (iii) the binding activity of RLSB (PBR1) to 5′-UTR of the *rbcL* mRNA is non-specific (Supplementary Figure S9B and C), (iv) more importantly, we have provided compelling evidence that the growth defects of the *pbr1-1* mutant plants can be rescued to the wild-type levels by allotopic expression of *Ycf1* in the nucleus (Figure 7). If *rbcL* was the major target of PBR1 in *Arabidopsis*, we would not count on sufficiently rescuing the *pbr1-1* mutant plants by relocation of *Ycf1* in the nucleus. One of the possibilities for this discrepancy is that, in Bowman's studies, most of experiments were performed using the *rlsb*-silenced plants rather than the T-DNA insertion lines because the line SALK_015722 that they isolated did not stably maintain the T-DNA insert [46]. By contrast, two T-DNA insertion lines SALK_124725 (knockdown line, designated as *pbr1-1*) and SALK_107226C (knockout line, designated as *pbr1-2*) were used in our studies and transforming genomic fragments of *PBR1* complemented the growth defects of both *pbr1-1* and *pbr1-2* mutants. The second possibility could be that the *RLSB* (*PBR1*)-silenced plants were initially selected in a kanamycin-containing medium while the control wild-type plants were grown on the same medium except without the initial selection, which may result in the difference in the level of the RbcL protein between two genotypes.

A key point of this study is that we identified Ycf1 as the direct downstream target protein of PBR1 by measuring the rate of translation of the chloroplast-encoded polypeptides (Figure 5, Supplementary Figure S7 and Supplementary Table S1) and examining polysomal association of the *Ycf1* mRNA between wild-type and *pbr1-1* mutant (Figure 6). As one of the two giant open reading frames in the chloroplast genome, *Ycf1* has been suggested to correspond to a pseudogene that has lost function [51–53]. However, the knockout of *Ycf1*, potentially specifying a protein of 1901 amino acids, yields chloroplast transformants but does not give rise to homoplastomic plants, implying that *Ycf1* is required for cell survival [35]. In *Chlamydomonas*, *orf1995* shares a low degree of similarity with *Ycf1* in tobacco and is also indispensable for cell survival [54]. In this study, we unexpectedly found that PBR1-modulated translation of *Ycf1* controls the concerted biogenesis of NDH, PSI and Cyt*b*_6_*f* complexes. Recently, Ycf1 has been shown to form a translocon complex with three other essential components in the inner-envelope membrane of chloroplasts, termed TIC [55]. In this study, we provided several lines of evidence that Ycf1 contributes to the concerted biogenesis of NDH, PSI and Cyt*b*_6_*f* complexes in *Arabidopsis*. It is reasonable to conclude that Ycf1 functions both in thylakoid membranes and the inner-envelope membrane of chloroplasts because (i) the Ycf1 protein was detected in the bands representing the NDH–PSI supercomplex, PSI and PSII core dimers and Cyt*b*_6_*f* complex (Figure 6a and b), (ii) Ycf1 was identified in the band corresponding to the NDH–PSI supercomplex by liquid chromatography–mass spectrometry (LC-MS/MS) on a high-performance mass spectrometer (Figure 5b and Supplementary Table S1), (iii) allotopic expression of *Ycf1* in the nucleus sufficiently rescues defects in the biogenesis of NDH, PSI and Cyt*b*_6_*f* complexes in *PBR1*-deficient plants (Figure 7) and (iv) the CC domains of Ycf1 interact with the representative subunits of PSI and Cyt*b*_6_*f* complexes and a known thylakoid protein Ycf4 [12] in a SUY2H system (Supplementary Figure S11B). These results strongly suggest that Ycf1 has a dual-localization pattern and is targeted to both the thylakoid membrane and inner-envelope membrane of chloroplasts. Indeed, the localization of Tic20 proteins is also not restricted to the inner-envelope membrane of chloroplasts, indicating that Tic proteins may have roles independent of translocon functions [56]. For instance, Tic62, a subunit of the Tic complex, is also localized in thylakoids and forms a high molecular weight complex with ferredoxin-NADP(H) oxidoreductase [57]. Although we cannot rule out the possibility that import of other, as yet uncharacterized, nuclear-encoded regulator proteins could be affected in the *pbr1-1* mutant, several lines of evidence presented in this study led us to favor the model that PBR1 modulates the translational efficiency of Ycf1 that functions as a platform in thylakoid membranes in selectively controlling photosynthetic complex biogenesis in higher plants.

In most land plant lineages, the Ycf1 gene is likely to have elevated substitution rates and evolved dramatically along with the evolution of the green lineage [58, 59]. In accordance with the hypothesis mentioned above, there is a large degree of Ycf1 sequence variability in annotated genomes (particularly among the algae), indicating a more complicated evolutionary history. The loss of Ycf1 from the plastomes of some (but not all) derived monocot lineages such as grasses, however, raise the question whether it can really carry out essential functions in all plants [60, 61].

In this study, we have demonstrated that PBR1 functions as a translational activator of Ycf1, serving to control biogenesis of NDH, PSI and Cyt*b*_6_*f* complexes in *Arabidopsis*. We have also proposed that Ycf1 may function in two possible ways: (i) Ycf1 may have a role as a scaffold protein recruiting the subunits of the photosynthetic complexes; and (ii) Ycf1 may be involved in the assembly process of the photosynthetic complexes as it also interacts with the assembly factor Ycf4, which was detected in the complex of PSI [12]. Considering that PBR1 is in all kinds of green organisms, including grasses (Figure 2), so how does the PBR1–Ycf1 module works in grasses? Given that nuclear-encoded and plastid-targeted proteins similar to Ycf1 were not found in grasses [60–62], one of explanations to address this puzzling problem is that there might be an alternative grass-type noncanonical homolog of Ycf1, whose translation is regulated by PBR1. Another possibility is that loss of Ycf1 might in fact point toward the function of PBR1 decoupled from controlling biogenesis of the indicated photosynthetic complexes in at least grasses, in which a PBR1-independent alternative regulatory mechanism has evolved. For instance, putatively functional copies of rbcL are retained in several representatives of non-photosynthetic plants [62]. Taken together, understanding the regulatory role of Ycf1 in the concerted biogenesis of photosynthetic complexes will require more investigations in future.

Although many of the nuclear-encoded *trans*-factors governing the biogenesis of NDH, PSI or Cyt*b*_6_*f* complexes have been identified, prior to this study, it is not known whether the biogenesis processes of these three complexes are finely coordinated. Remarkably, the nuclear-encoded PBR1 tightly controls the expression of the chloroplast gene *Ycf1* at the translational level, which is sufficient to sustain the coordinated biogenesis of NDH, PSI and Cyt*b*_6_*f* complexes as a whole. Our genetic analyses illustrating the essential nature of the PBR1–Ycf1 module in selective control of NDH, PSI and Cyt*b*_6_*f* biogenesis provide deep insights into better understanding as to how a predominant nuclear-encoded factor can act as a migratory mediator and undergoes translational regulation of the target plastid gene in the chloroplast in controlling biogenesis of photosynthetic complexes.

## Materials and Methods

### Plant material and growth conditions

Wild-type and mutant *Arabidopsis thaliana* plants (ecotype Columbia) were grown in a growth chamber or a phytotron under long day conditions, 16 h of white light (80 μmol m^−2^s^−1^) and 8 h of dark at 21 °C. Seeds were grown in peat soil culture without sterilization or surfaced-sterilized, plated on half-strength MS medium supplemented with different concentrations of sucrose and stratified at 4 °C for 3 days. The *pbr1-1* (SALK_124725) and *pbr1-2* (SALK_107226C) mutants were obtained from the Arabidopsis Biological Resource Center (ABRC, Columbus, OH, USA). The *pbr1* mutants were backcrossed to wild type twice for removing background mutations.

### Generation of transgenic plants

For the genomic complementation, a genomic fragment of *PBR1* (At1g71720, 5152 bp in size), starting at 2 598 bp upstream of the ATG codon and ending at 404 bp after the stop codon, was amplified from genomic DNA by PCR with GC-F and GC-R primers, digested with *Bam*HI and *Xho*I and cloned into *Bam*HI and *Sal*I sites of the pCAMBIA1300 binary vector. To generate the transgenic plants overexpressing *PBR1*, a cDNA clone containing the full-length *PBR1* open reading frame was amplified by PCR with OE-F and OE-R primers and inserted into *Sal*I and *Sma*I cloning sites in the 35S CaMV expression cassette of the pBIN-JIT vector. For the expression pattern analysis, a promoter fragment extending 2 598 to 1 bp upstream of the translation initiation ATG codon of *PBR1* was amplified using the primers GUS-F and GUS-R and the resulting fragment was cloned into *Hind*III and *Bam*HI sites of the binary vector pBI101.1 to yield the *Pro-PBR1::GUS* construct. For subcellular analysis, a cDNA clone containing the full-length *PBR1* open reading frame was amplified by PCR with YFP-F and YFP-R primers, digested with *Bam*HI and *Xho*I and inserted into *Bgl*II and *Xho*I cloning sites of the pMON530-eYFP-G vector, yielding a C-terminal YFP fusion construct. For allotopic expression of the chloroplast gene *Ycf1* in the nucleus in the *pbr1-1* mutant, the full-length cDNA of *Ycf1* was generated by linking three fragments of *Ycf1* cDNA (A, B and C), which were amplified by PCR with three primer pairs (Ycf-A-F, Ycf-A-R; Ycf-B-F, Ycf-B-R; Ycf-C-F, Ycf-C-R), respectively. First, the A-fragment (1 360 bp from the start codon to the *Hind*III restriction site) was amplified using the primer pair Ycf-A-F and Ycf-A-R with the introduction of *Sal*I and *Nhe*I restriction sites at the 5′ terminus and *Xba*I and *Sma*I sites at the 3′ terminus and cloned into the pMD-19T simple vector (Takara, Dalian, China). The resulting construct was named as Ycf-A-PMD-19T. The *Sal*I and *Nhe*I restriction sites at the 5′ terminus were designed as the cloning sites for inserting the plastid-transit peptide sequence (240 bp) from the *RbcS* gene, encoding the small subunit of Rubisco [63]. The *Xba*I and *Sma*I sites at the 3′ terminus, which are immediately following the *Hind*III site, were designed as restriction sites for subcloning the B and C fragments of the *Ycf1* cDNA subsequently. Second, the B fragment (2 626 bp from the *Hind*III site to the *Xba*I site) was amplified with primers Ycf-B-F and Ycf-B-R. The amplified B-fragment was subcloned into Ycf-A-PMD-19T with *Hind*III and *Xba*I sites to yield the Ycf-A-B-PMD-19T. Third, the C fragment (1,384 bp from the *Xba*I site to the stop codon) was amplified using the primers Ycf-C- F and Ycf-C- R with the introduction of *Sma*I site at the 3′ terminus, immediately following the stop codon. The amplified C-fragment was subcloned into Ycf-A-B-PMD-19T with *Xba*I and *Sma*I sites to yield the Ycf-A-B-C-PMD-19T. Next, the full-length cDNA of *Ycf1*, released from the the Ycf-A-B-C-PMD-19T, was subcloned into pBIN-JIT vector with *Sal*I and *Sma*I cloning sites to yield the Ycf1-pBIN-JIT. Finally, the amplified fragment (RbcS-TP) of the plastid-transit peptide sequence (240 bp) from the *RbcS* gene was subcloned into the resulting Ycf1-pBIN-JIT construct with the *Sal*I and *Nhe*I cloning sites to yield the RbcS-TP-Ycf1-pBIN-JIT. The primer sequences are listed in Supplementary Table S2.

*Agrobacterium tumefaciens* strain GV3101, harboring the desired constructs, was used to transform plants with a floral dip method. Transgenic plants were screened on solid plates containing 50 mg l^−1^ kanamycin or 25 mg l^−1^ hygromycin.

### Quantitative real-time RT–PCR and RT–PCR

Total RNA was isolated from samples frozen in liquid nitrogen using the RNAiso Plus (Takara) according to manufacturer’s protocol. For quantitative real-time RT–PCR and RT–PCR analysis, DNA contaminated in total RNA samples was digested with RNase-free DNaseI (Takara). Complementary DNA was generated using an oligo d(T)18 primer (Takara) or random primers (Toyobo, Osaka, Japan) for the chloroplast gene *Ycf1* [64]. Quantitative real-time PCR was performed with SYBR Premix Ex TaqII (Takara) using a MyiQ5 single color Real-Time PCR Detection System (Bio-Rad, Hercules, CA, USA) as described [65]. Primer names and sequences are listed in Supplementary Table S2.

### Transmission electron microscopy

Sample fixation and examination were performed as described [65]. Samples were fixed with 2.5% (v/v) glutaraldehyde and 2% (v/v) paraformaldehyde. A transmission electron microscope (H-7650, Hitachi, Tokyo, Japan) was employed to examine thin sections using a voltage of 80 kV.

### Analysis of photosynthetic complexes in thylakoid membranes

Thylakoid membranes were prepared as described previously [66]. For one-dimensional electrophoresis, thylakoid membranes were separated by SDS-PAGE and western blots were performed as described previously [65]. The isolated thylakoid membranes were separated by BN-PAGE as described [66, 67]. For two-dimensional SDS-PAGE separation, the excised BN-PAGE lanes were denatured in SDS sample buffer and 5% β-mercaptoethanol and layered onto 12% SDS polyacrylamide gels with 4 M urea. For immunodetection of thylakoid membrane proteins, the separated proteins were transferred to nitrocellulose membranes and probed with the indicated specific antibodies (Agrisera, Vännäs, Sweden). For detecting signals, alkaline-phosphatase-conjugated goat anti-rabbit IgG (Millipore, Darmstadt, Germany) was used as a secondary antibody, and reactions were revealed using an ECL Kit (GE Healthcare, Marlborough, MA, USA). The signals were detected by ImageQuant LAS 4000 mini (GE Healthcare).

### Analysis of chlorophyll fluorescence

The measurements of chlorophyll fluorescence were performed using CF Imager (Technologica, Essex, UK) as described in the manufacturer’s instructions. The preprogammed regimes of AL exposure times, saturating light pulses and imaging of the parameters Fv′/Fm′, NPQ, Φ (PSII) and qP were performed automatically by CF Imager and the associated software. After transfer of the darkness-adapted plants into the CF Imager cabinet, AL (600 μmol m^−2^ s^−1^) was switched on and saturating pulses of light (6000 μmol m^−2^ s^−1^) were applied automatically once every 30 s in 5 min. The intensities for ALs and saturating pulses of light were chosen as in Mishra *et al*. [68] with modifications. Image data acquired in each experiment were normalized to a false-color scale with arbitrarily assigned extreme values.

The activity of PSI was monitored via leaf absorbance changes at 820 nm using a Walz PAM-101 system connected to a dual-wavelength P700 unit (ED-P700DW, WALZ, Effeltrich, Germany) [69, 70]. Switching on far-red light (720 nm) was used to oxidize P700 and a saturating pulse of white light was then applied to reduce P700. The redox levels of P700 were normalized to leaf area. Leaf areas were measured by Image J (NIH, Bethesda, MD, USA). The NDH activity was monitored by a transient increase in chlorophyll fluorescence when AL (50 μmol of photons m^−2^ s^−1^) was switched off [30, 71].

### SDS-PAGE electrophoresis and immunoblot analysis

Total protein was extracted as described [72]. SDS-PAGE electrophoresis and western blots were performed as described previously [65]. Reactions were revealed using an ECL Kit (GE Healthcare), and signals were visualized by ImageQuant LAS 4000 mini (GE Healthcare). Anti-PBR1 or Ycf1 rabbit polyclonal antisera were generated against the peptide of PBR1 (from Ile^400^ to Ser^499^) or the peptide of Ycf1 (from Lys^1673^ to Pro^1785^) by the Abmart Biomedical Company (Shanghai, China) and CoWin Biotech Company (Beijing, China), respectively. Antisera against α-Tublin and RbcL were purchased from Sigma-Aldrich (St Louis, MO, USA) and Agrisera, respectively.

### In vivo chloroplast protein translation assay

*In vivo* chloroplast protein labeling was performed as described previously [45, 65]. For comparative analysis, the leaves of wild type and *pbr1-1* or overexpression line of *PBR1* (OE-5) were pulse-labeled with 200 mg l^−1^ ^13^C_6_^15^N_4_ L-Arginine (‘heavy’, H) and ^15^N_4_ L-Arginine (‘medium heavy’, M), respectively. Thylakoid membranes were extracted from the labeled leaves as described [66]. The extracted thylakoid membranes were separated by BN-PAGE as described [66, 67]. Gel slices corresponding to NDH–PSI supercomplex [30] were excised, and the extracted peptides were analyzed by LC-MS/MS on a high-performance mass spectrometer (LTQ-Orbitrap XL,Thermo Finnigan, San Jose, CA, USA) as described previously [65]. Raw data were processed using the MaxQuant 1.1.36 software package (http://www.maxquant.org/) for protein identification and quantitation.

### Polysome purification and northern blots

Polysomes were isolated as described previously [73]. Briefly, polysomes isolated from equal fresh weight of leaves were fractionated in linear 15–55% sucrose gradients by ultracentrifugation. Ten fractions with equal volume were collected from top to bottom of the sucrose gradients. RNA was purified from each fraction by detergent treatment, phenol extraction and ethanol precipitation. RNA samples were fractionated on 1.2% (w/v) agarose gel containing formaldehyde and then transferred onto a Hybond-N^+^ membrane (GE Healthcare). The blotted membrane was hybridized with the indicated DIG-labeled gene-specific probes in DIG Easy-Hyb solution (Roche) at 65 °C for 16 h. DIG Probe synthesis Kit (Roche) was used for synthesizing the gene-specific probes by PCR. After hybridization, the resulting membrane was washed according to the manufacturer’s protocols. After washing, the membrane was further treated with DIG Wash and Block Buffer Set (Roche) and then incubated with anti-digoxigenin conjugated to alkaline phosphatase (Roche) according to the manufacturer’s protocols. Hybridization signals were developed with CDP-Star (Roche) and visualized by the ImageQuant LAS4000 mini system (GE Healthcare). The related primer sequences are listed in Supplementary Table S2.

### Yeast two-hybrid assay

NubG and Cub are able to reconstitute ubiquitin in the split-ubiquitin assay only when brought into close proximity by two interacting test proteins that are expressed as fusion proteins with NubG and Cub. The yeast two-hybrid assay was performed as described previously [74]. The mature full-length coding regions of the *psaA*, *psaB*, *petA*, *atpB* and *Ycf4* genes were amplified by PCR and cloned into the vector of PMD-19T simple (Takara) and then sequenced. The sequence-confirmed fragments were cloned into the bait vector *Cub-PLV* vector, *pMetYCgate*. The sequenced coding fragments of the CC domains (CC1 and CC2) of Ycf1 were amplified and then cloned into the prey *Nub* vector, *pNXgate32*. The resulting *Nub* and *Cub* constructs were cotransformed into the yeast strain *THY.AP4*. Cotransformants were selected on synthetic medium lacking Leu and Trp. The interaction was selected on synthetic medium lacking Leu, Trp and His. We are grateful to Dr Yong-Fei Wang for providing us the bait and prey vectors. The related primer sequences are listed in Supplementary Table S2.

### Expression and purification of recombinant PBR1 protein

The full-length *PBR1* open reading frame was amplified by PCR with RC-F and RC-R primers and inserted into the *Bam*HI and *Eco*RI cloning sites of pET28-a vector. The extracted 6xHis-PBR1 recombinant fusion proteins from the *Escherichia coli* BL21 (DE3) strain were purified with Ni-NTA resin (Qiagen, Valencia, CA, USA).

### Electrophoretic mobility shift assay

The 5′- and 3′-UTR fragments of the *Ycf1* cDNA were amplified using the primer pairs (Ycf-5′UTR-F and Ycf-5′UTR-R; Ycf-3′UTR-F and Ycf-3′UTR-R), respectively, with the introduction of the T7 promoter sequence at the 5′ terminus and *Xho*I site at the 3′ terminus. The resulting fragments were cloned into pMD-19T simple vector (Takara), and the resulting construct was subsequently linear with the *Xho*I digestion to be used as templates for next-step probe generation. The biotin-labeled RNA probes were generated using T7 RNA polymerase (Thermo Scientific, Waltham, MA, USA) by adding biotin-16-UTP (Roche) in the reaction mixture. For generating ‘cold’ probes, the same reactions were conducted by adding non-labeled UTP. EMSA was performed with a Light Shift Chemiluminescent RNA EMSA kit (Thermo Scientific) according to the manufacturer’s instruction. For the binding reactions, recombinant PBR1 proteins and labeled RNA probes were incubated on ice for 30 min in the binding buffer (20 mM HEPES, pH 8.0, 50 mM KCl, 10 mM MgCl_2_, 0.5 mM dithiothreitol, 0.2 μg μl^−1^ tRNA, 0.1 μg μl^−1^ bovine serum albumin (Fraction V) and 4% (v/v) glycerol). Non-labeled RNA probes were used as competitor. Free and bound probes were separated by 6% native PAGE in 0.5× TBE buffer (4 °C) at 50 V for 0.5 h and subsequently at 80 V for 2 h. The gel was then transferred to a Hybond-N^+^ membrane (GE Healthcare) with a Trans-Blot SD semi-dry transfer cell (Bio-Rad). The blotted free and bound probes were cross-linked to the membrane under ultraviolet light for 0.8 min. The membrane was treated with developing buffers according to the manufacturer’s instruction. The signals were detected by ImageQuant LAS 4000 mini (GE Healthcare).

### Confocal microscopy and fluorescence microscopy

YFP images were visualized by a FLUO-VIEW FV1000 laser scanning confocal microscopy (Olympus, Tokyo, Japan) or a BX51 fluorescence microscopy (Olympus). YFP signals were excited at 488 nm, and emissions were collected with a 500- to 600-nm band-pass filter. Chlorophyll autofluorescence was excited at 635 nm, and emissions were collected with a 650–750-nm band-pass filter.

### GUS staining

The etiolated transgenic seedlings were stained as previously described [65].

### Bioinformatics analysis

Amino-acid sequences of the PBR1 homologs were retrieved using the BLAST algorithm (http://blast.ncbi.nlm.nih.gov/Blast.cgi). Multiple sequence alignments were performed using the ClustalW program (http://www.genome.jp/tools/clustalw/). Sequence alignments for presentation were colored using Boxshade (http://www.ch.embnet.org/software/BOX_form.html). S1 RNA-binding domains were predicted using Pfam (http://pfam.sanger.ac.uk/). A Neighbor-Joining tree was constructed using MEGA6 (http://www.megasoftware.net/) with bootstrap test for 1 000 replicates. The tree was drawn using the FigTree program (http://tree.bio.ed.ac.uk/software/figtree/). The CC domains of Ycf1 were predicted by the COILS program (http://www.ch.embnet.org/software/COILS_form.html).

### Accession numbers

Sequence data from this article can be found in the Arabidopsis Genome Initiative or GenBank databases under the following accession numbers: *PBR1* (At1g71720), *Ycf1* (AtCG01130), *RBCS* (At1g67090), *ACTIN2* (At3g18780), GmPBR1 (XP_003535559), SlPBR1 (XP_004253265), ZmPBR1 (DAA58739), BdPBR1 (XP_003569284) and OsPBR1 (NP_001043440).
