# Supplementary material for: PBR1 selectively controls biogenesis of photosynthetic complexes by modulating translation of the large chloroplast gene Ycf1 in Arabidopsis
Source: Cell Discov. 2016 May 10;2:16003–. doi: 10.1038/celldisc.2016.3 (PMC4870678; doi:10.1038/celldisc.2016.3)
Supplement: Supplementary Figure S1 [file celldisc20163-s1.pdf]

Supplementary Information

Figure S1

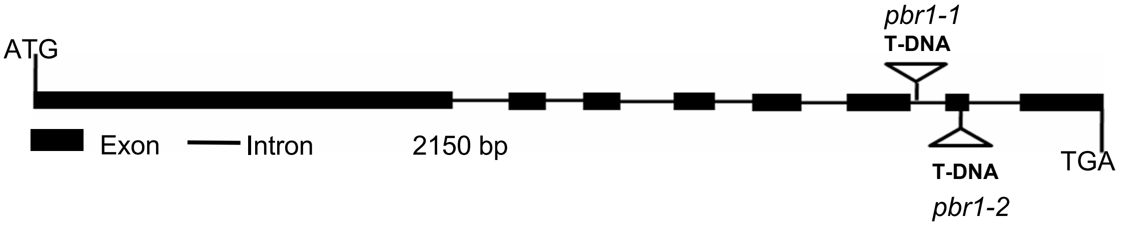

Figure S1 Schematic diagram of *PBR1* gene showing the T-DNA insertion sites. Closed box indicates ORF. Exons (boxes) and introns (lines) were determined by a comparison of the genomic and cDNA sequences. The T-DNA insertion sites and positions of the start and stop codons are indicated.
