# Supplementary material for: PBR1 selectively controls biogenesis of photosynthetic complexes by modulating translation of the large chloroplast gene Ycf1 in Arabidopsis
Source: Cell Discov. 2016 May 10;2:16003–. doi: 10.1038/celldisc.2016.3 (PMC4870678; doi:10.1038/celldisc.2016.3)
Supplement: Supplementary Figure S2 [file celldisc20163-s2.pdf]

Figure S2

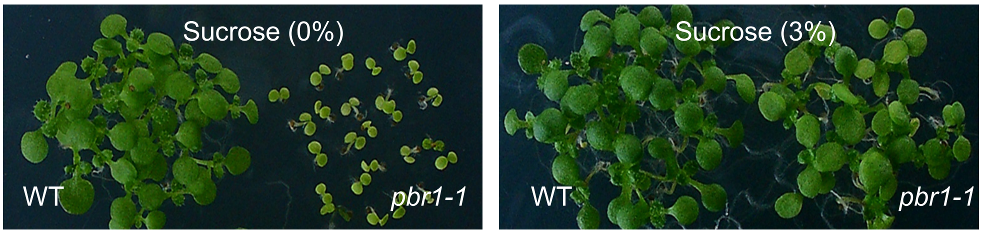

Figure S2 Supplementation of sucrose rescues growth defects of *pbr1-1.*
Phenotypes of 10-d-old seedlings of the wild-type, *pbr1-1* seedlings grown on ½ MS medium supplemented with 3% or without sucrose.
