# Supplementary material for: PBR1 selectively controls biogenesis of photosynthetic complexes by modulating translation of the large chloroplast gene Ycf1 in Arabidopsis
Source: Cell Discov. 2016 May 10;2:16003–. doi: 10.1038/celldisc.2016.3 (PMC4870678; doi:10.1038/celldisc.2016.3)
Supplement: Supplementary Figure S3 [file celldisc20163-s3.pdf]

Figure S3

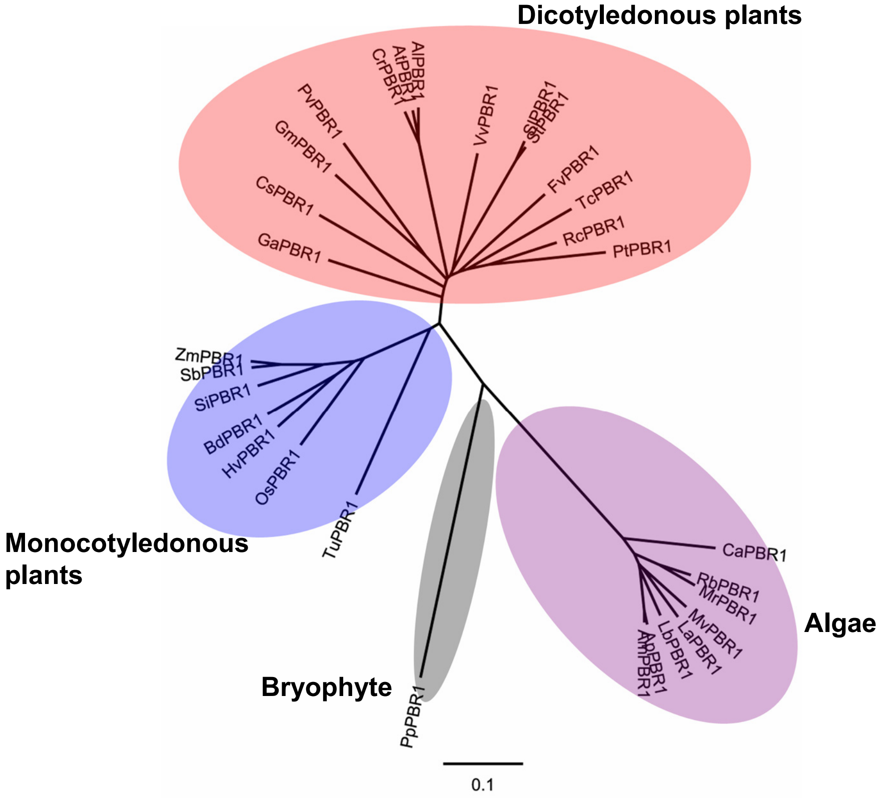

Figure S3 Phylogenetic relationships of PBR1 homologs.
Neighbor-Joining phylogenetic tree of PBR1 homologs from 30 species, including algae, bryophyte, monocotyledonous and dicotyledonous plants was generated with MEGA 6. Bootstrap analysis was conducted with 1000 replicates. A radial tree was drawn with FigTree. The bar indicates substitutions per residue. *Al, Arabidopsis lyrata* （ GenBank accession number:XP_002887384); *Am, Arthrospira maxima* （WP_006668626); *Ap, Arthrospira platensis* （YP_005072908); *At, Arabidopsis thaliana* （AT1G71720); *Bd,Brachypodium distachyon* (XP_003569284); *Ca, Cyanobacterium aponinum* (YP_007162445); *Cr, Capsella rubella* (EOA34039); *Cs, Cucumis sativus* (XP_004140950); *Fv, Fragaria vesca* (XP_004290474); *Ga, Genlisea aurea* (EPS66517); *Gm, Glycine max* (XP_003535559); *Hv, Hordeum vulgare* (BAJ92399); *La, Lyngbya aestuarii* (WP_023067953); *Lb, Leptolyngbya boryana* (WP_017290078); *Mr, Mastigocladopsis repens* (WP_017316016); *Mv, Microcoleus vaginatus* (WP_006635639); *Os, Oryza sativa* (NP_001043440); *Pp, Physcomitrella patens* (XP_001758864); *Pt, Populus trichocarpa* (XP_002302157); *Pv, Phaseolus vulgaris* (ESW23949); *Rb, Raphidiopsis brookii* (WP_009341336); *Rc, Ricinus communis* (XP_002527086); *Sb, Sorghum bicolor* (XP_002458071); *Si, Setaria italica* (XP_004969038); *Sl, Solanum lycopersicum* (XP_004253265); *St, Solanum tuberosum* (XP_006359028); *Tc, Theobroma cacao* (EOY16872); *Tu, Triticum urartu* (EMS62964); *Vv, Vitis vinifera* (XP_002263508); *Zm, Zea mays* (DAA58739).
