# Supplementary material for: PBR1 selectively controls biogenesis of photosynthetic complexes by modulating translation of the large chloroplast gene Ycf1 in Arabidopsis
Source: Cell Discov. 2016 May 10;2:16003–. doi: 10.1038/celldisc.2016.3 (PMC4870678; doi:10.1038/celldisc.2016.3)
Supplement: Supplementary Figure S4 [file celldisc20163-s4.pdf]

Figure S4

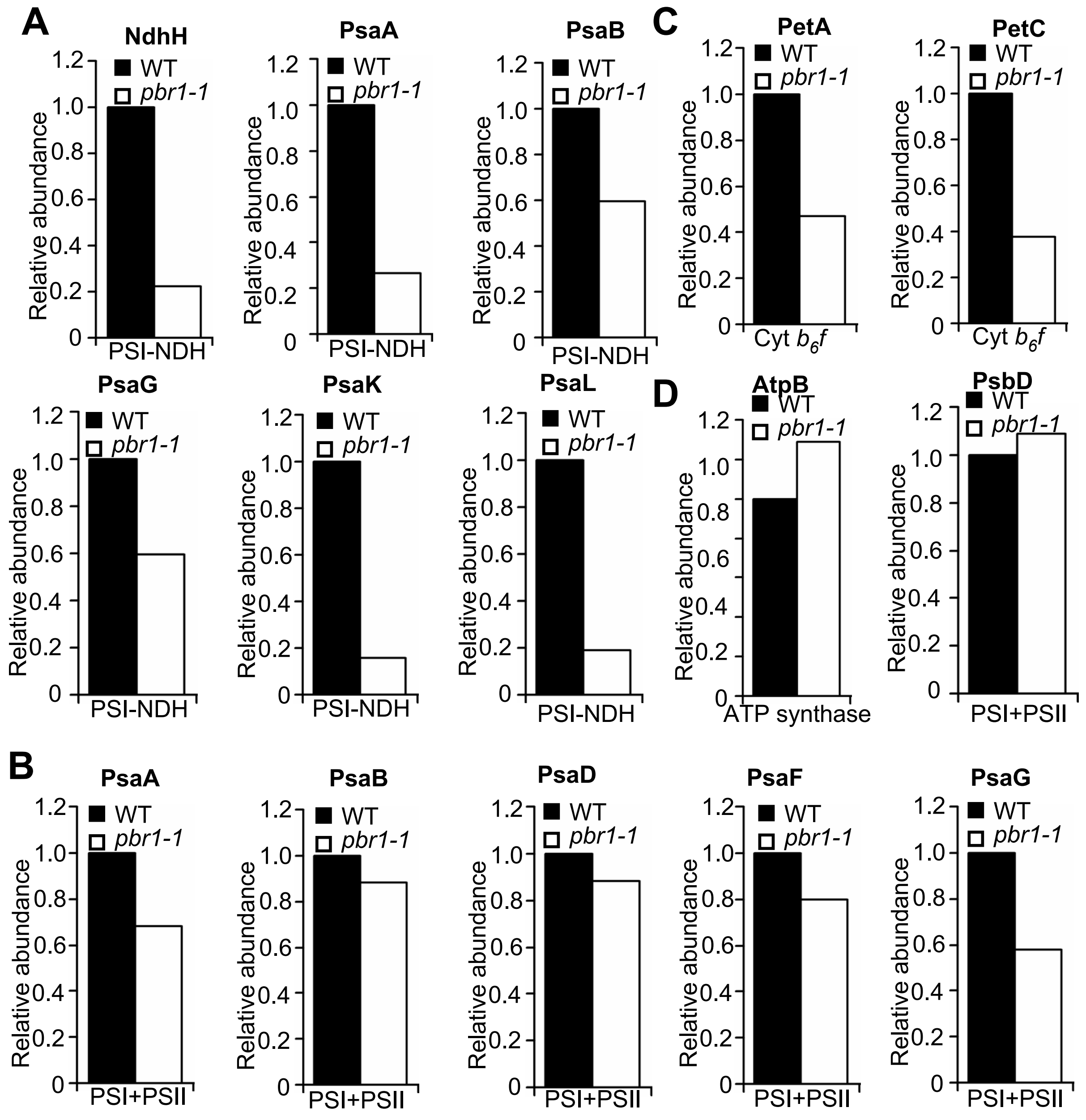

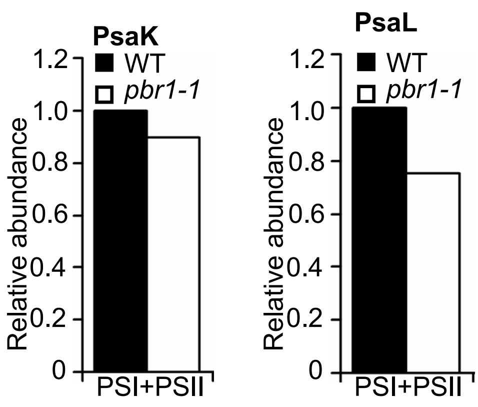

Figure S4 Intensity quantification of the western blot analysis in Figure 3B. Relative abundance of the indicated subunits in (**A**) PSI-NDH, (**B**) PSI+PSII core dimmer, (**C**) Cytb6/f complex, (**D**) AtpB and PsbD. Signal intensities were quantified with ImageQuant TL.
