# Supplementary material for: PBR1 selectively controls biogenesis of photosynthetic complexes by modulating translation of the large chloroplast gene Ycf1 in Arabidopsis
Source: Cell Discov. 2016 May 10;2:16003–. doi: 10.1038/celldisc.2016.3 (PMC4870678; doi:10.1038/celldisc.2016.3)
Supplement: Supplementary Figure S5 [file celldisc20163-s5.pdf]

Figure S5

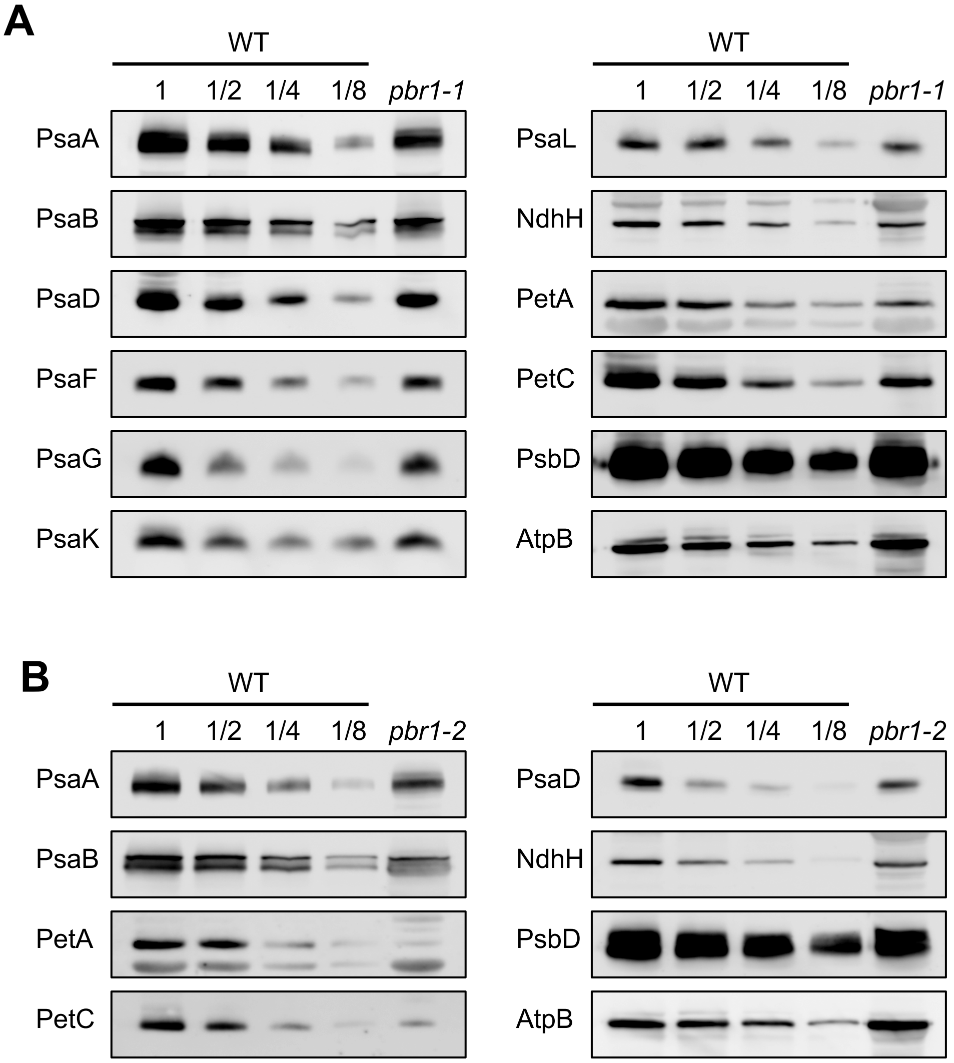

Figure S5 *PBR1* knockdown leads to reduction in abundance of NDH, PSI and Cyt*b6f* complexes.
(**A,B**) Comparative analysis of abundance of thylakoid complex proteins between *the pbr1-1* mutant and wild-type (A) or between *the pbr1-2* mutant and wild-type (B) by immunodetection with a wild-type dilution series (1/2, 1/4 and 1/8). Thylakoid membrane proteins were fractionated by SDS-PAGE and the resulting blots were probed with antibodies against the indicated proteins, respectively.
