# Supplementary material for: PBR1 selectively controls biogenesis of photosynthetic complexes by modulating translation of the large chloroplast gene Ycf1 in Arabidopsis
Source: Cell Discov. 2016 May 10;2:16003–. doi: 10.1038/celldisc.2016.3 (PMC4870678; doi:10.1038/celldisc.2016.3)
Supplement: Supplementary Figure S6 [file celldisc20163-s6.pdf]

Figure S6

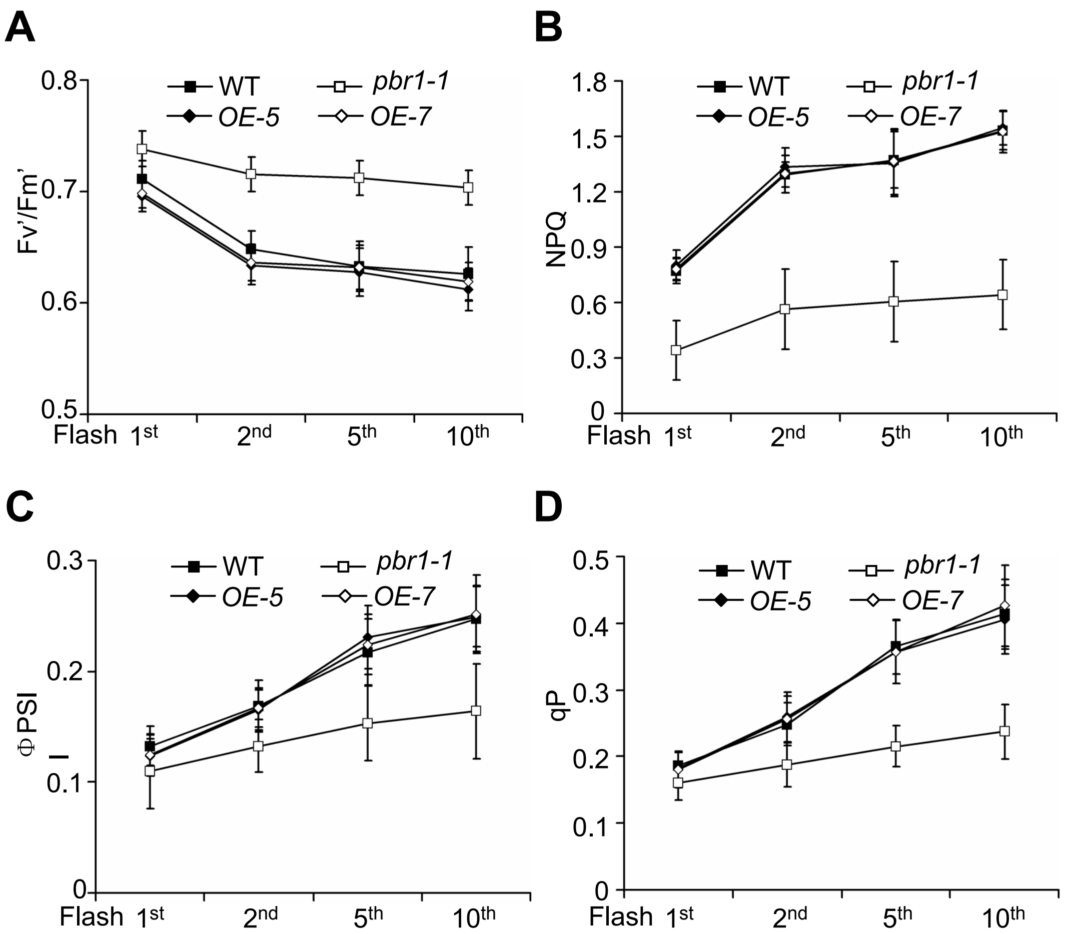

Figure S6 Comparison of pixel values of photosynthetic parameters within fluorescence ratio images Fv'/Fm',NPQ, ΦPSII and qP shown in Figure 4C. (**A-D**) Statistical analysis of the pixel values of photosynthetic parameters, including Fv'/Fm' (A), NPQ (B), ΦPSII (C) and qP (D), in leaves of the wild type, *pbr1-1* and *PBR1*-OE lines (OE-5 and OE-7) corresponding to the images shown in Figure 4c. The pixel values of photosynthetic parameters for each genotype were shown at the first, second, fifth and tenth flash, respectively. 50 representative pixel values of each photosynthetic parameter within fluorescence ratio images were taken at the first, second, fifth and tenth flash point for statistical analysis. Error bars indicate SD (n=50).
