# Supplementary material for: PBR1 selectively controls biogenesis of photosynthetic complexes by modulating translation of the large chloroplast gene Ycf1 in Arabidopsis
Source: Cell Discov. 2016 May 10;2:16003–. doi: 10.1038/celldisc.2016.3 (PMC4870678; doi:10.1038/celldisc.2016.3)
Supplement: Supplementary Figure S7 [file celldisc20163-s7.pdf]

Figure S7

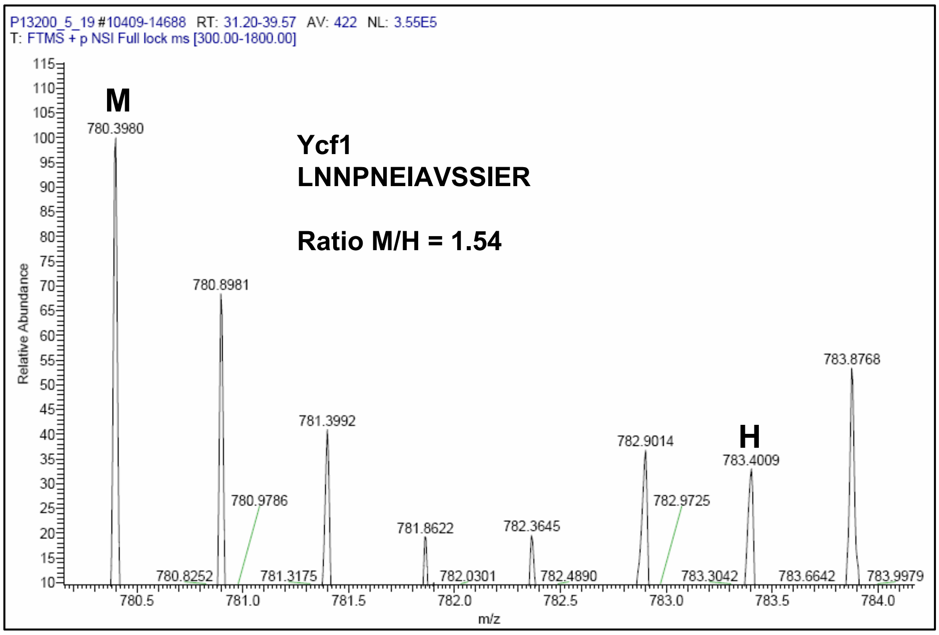

Figure S7 Overexpression of *PBR1* enhances the Ycf1 translation. The representative mass spectra for identification of Ycf1 protein in samples extracted from the wild-type and *PBR1*-overexpression line (OE5) leaves pulse-labeled with “heavy” (H) and “medium heavy” (M) stable isotope amino acids, respectively. The ratio of peak intensities of M versus H peptides reflects difference between the overexpression line OE5 and wild-type in translation of the corresponding proteins since the newly synthesized proteins incorporate either the M or H amino acids.
