# Supplementary material for: PBR1 selectively controls biogenesis of photosynthetic complexes by modulating translation of the large chloroplast gene Ycf1 in Arabidopsis
Source: Cell Discov. 2016 May 10;2:16003–. doi: 10.1038/celldisc.2016.3 (PMC4870678; doi:10.1038/celldisc.2016.3)
Supplement: Supplementary Figure S8 [file celldisc20163-s8.pdf]

Figure S8

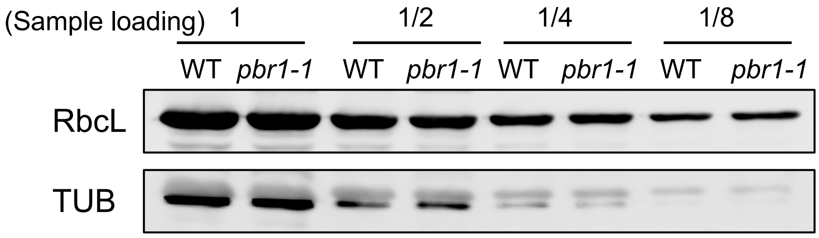

Figure S8 Effects of *PBR1* knockdown on the protein levels of RbcL. The protein levels of RbcL in leaves of the wild-type and *pbr1-1* mutant plants analyzed by western blots with an polyclonal antibody against RbcL. From left to right, the lanes of the upper blot show the undiluted wild-type (WT) and *pbr1-1* mutant protein samples (10 μg) and dilutions to 50, 25, 12.5 % of the WT and *pbr1-1* samples. Equal protein loading was confirmed with antiserum against α-Tubulin (TUB).
