# Supplementary material for: PBR1 selectively controls biogenesis of photosynthetic complexes by modulating translation of the large chloroplast gene Ycf1 in Arabidopsis
Source: Cell Discov. 2016 May 10;2:16003–. doi: 10.1038/celldisc.2016.3 (PMC4870678; doi:10.1038/celldisc.2016.3)
Supplement: Supplementary Figure S9 [file celldisc20163-s9.pdf]

Figure S9

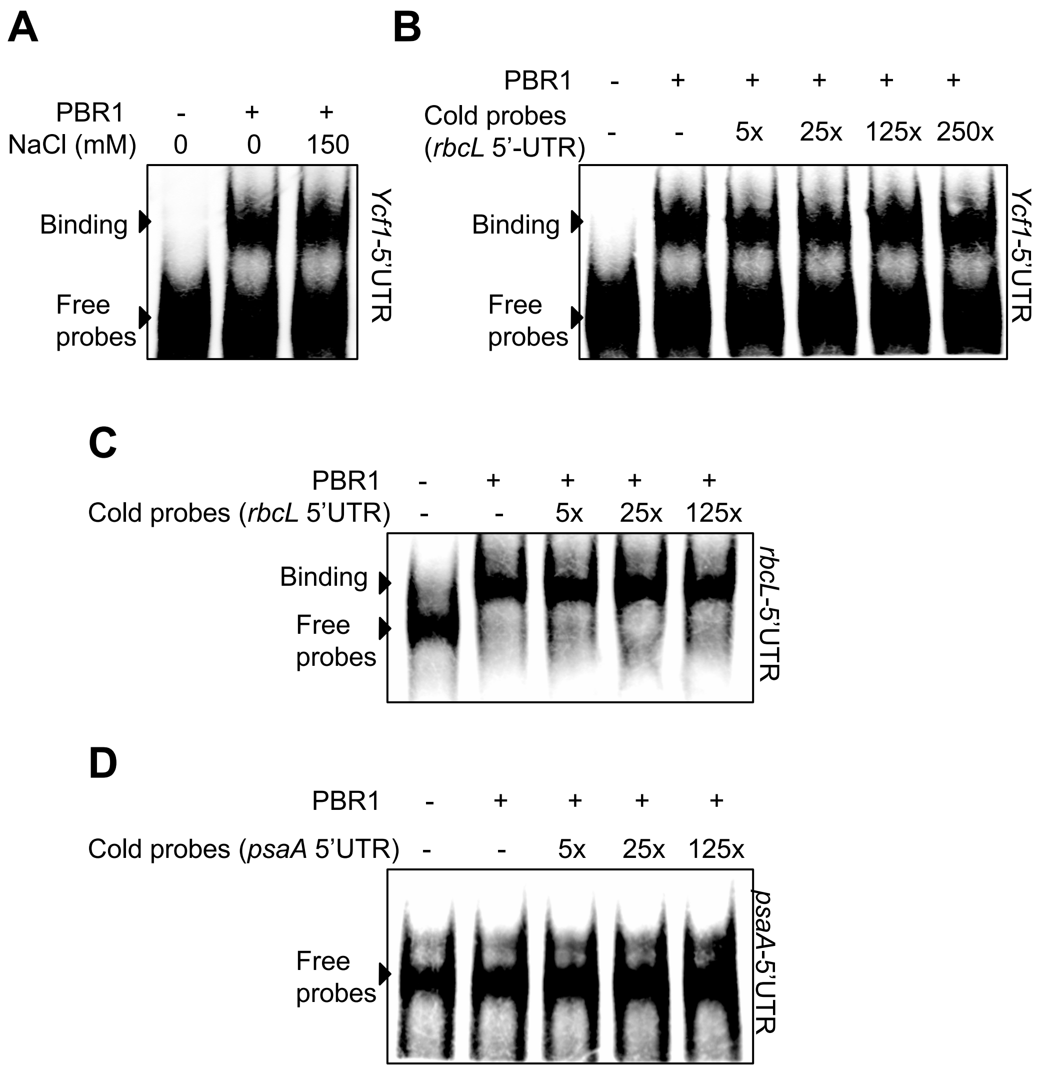

Figure S9 Binding specificity of PBR1 to 5'-UTR regions of the *Ycf1, rbcL* and *psaA* transcripts.
(**A**) Effect of high concentration of NaCl (150 mM) on the binding activity of PBR1 to 5'-UTR of the *Ycf1* mRNA.
(**B**) Binding activity of PBR1 to 5'-UTR of the *Ycf1* mRNA in the presence of increasing amounts of unlabelled 5'-UTR of *rbcL* mRNA as a cold oligonucleotide competitor.
(**C**) Binding activity of PBR1 to 5'-UTR of the *rbcL* mRNA in the presence of increasing amounts of unlabelled 5'-UTR of *rbcL* mRNA as a cold oligonucleotide competitor.
(**D**) Binding activity of PBR1 to 5'-UTR of the *psaA* mRNA in the presence of increasing amounts of unlabelled 5'-UTR of *psaA* mRNA as a cold oligonucleotide competitor.
