# Supplementary material for: PBR1 selectively controls biogenesis of photosynthetic complexes by modulating translation of the large chloroplast gene Ycf1 in Arabidopsis
Source: Cell Discov. 2016 May 10;2:16003–. doi: 10.1038/celldisc.2016.3 (PMC4870678; doi:10.1038/celldisc.2016.3)
Supplement: Supplementary Figure S10 [file celldisc20163-s10.pdf]

Figure S10

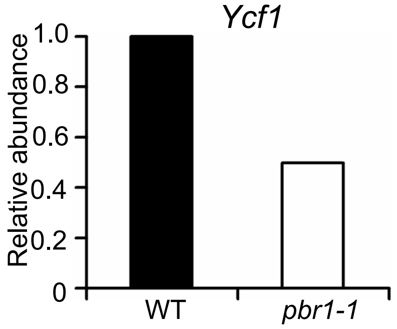

Figure 10 Quantification of the signal intensities for the northern blot analysis as shown in Figure 6C.

The signal intensities of Lane 9 from wild type and the *pbr1-1* mutant were quantified respectively with ImageQuant TL.
