# Supplementary material for: PBR1 selectively controls biogenesis of photosynthetic complexes by modulating translation of the large chloroplast gene Ycf1 in Arabidopsis
Source: Cell Discov. 2016 May 10;2:16003–. doi: 10.1038/celldisc.2016.3 (PMC4870678; doi:10.1038/celldisc.2016.3)
Supplement: Supplementary Figure S11 [file celldisc20163-s11.pdf]

Figure S11

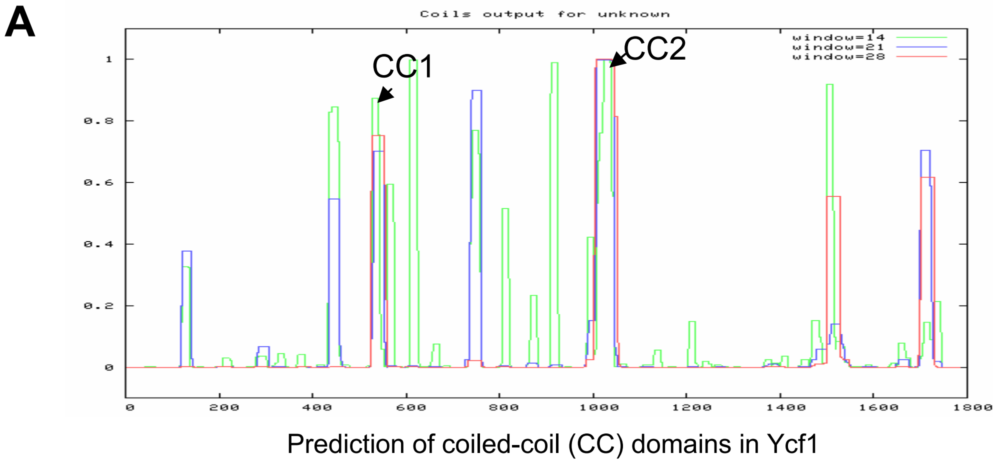

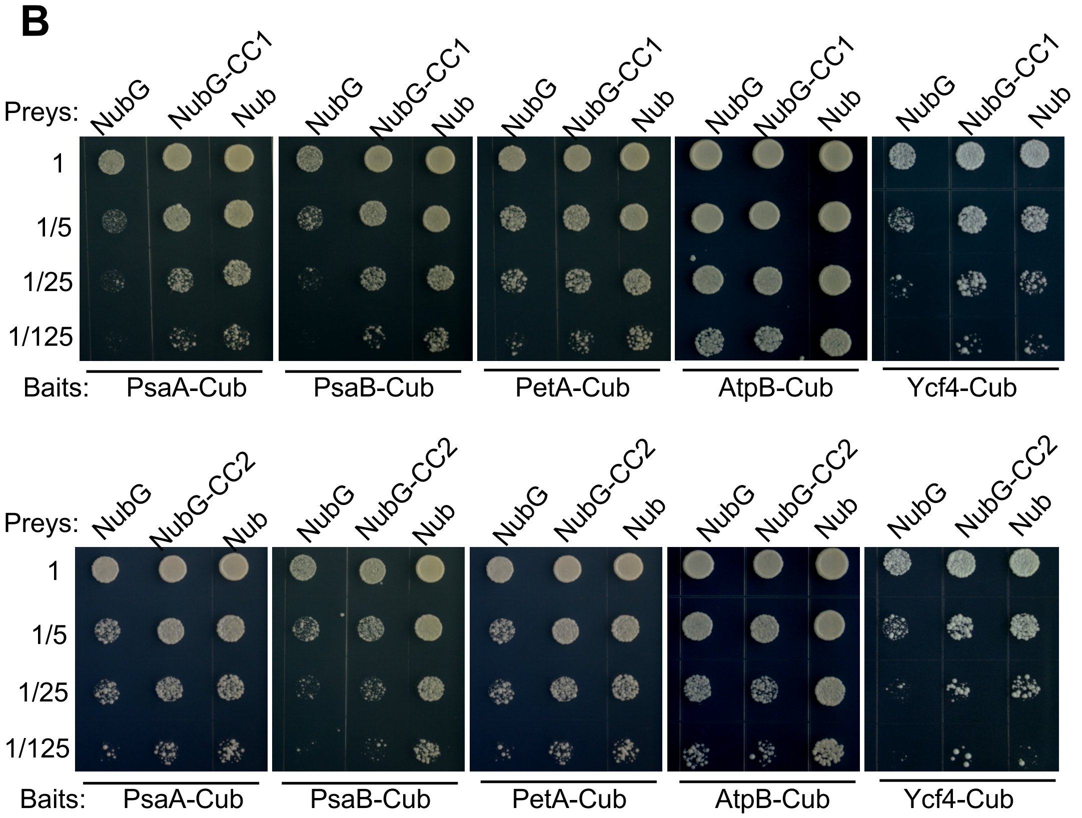

Figure S11 Coiled-coil domains of Ycf1 interact with subunits of PSI and Cytb_6f complexes and a thylakoid protein Ycf4 in a split-ubiquitin yeast two-hybrid (SUY2H) system.

(**A**) Two coiled-coil domains, designated as CC1 and CC2, in Ycf1 predicted by COILS program (http://www.ch.embnet.org/software/COILS_form.html)

(**B**) Interaction of coiled-coil domains (CC1 and CC2) of Ycf1 with the indicated subunits of PSI and Cytb_6f complexes and Ycf4. Yeast cells cotransformed with Cub-the indicated subunit or -Ycf4 as baits and NubG, NubG-CC1 or -CC2, and Nub as preys grown on stringent media lacking His, Leu, and Trp with a dilution series (1/5,1/25,1/125).
