# Supplementary material for: PBR1 selectively controls biogenesis of photosynthetic complexes by modulating translation of the large chloroplast gene Ycf1 in Arabidopsis
Source: Cell Discov. 2016 May 10;2:16003–. doi: 10.1038/celldisc.2016.3 (PMC4870678; doi:10.1038/celldisc.2016.3)
Supplement: Supplementary Table S1 [file celldisc20163-s12.pdf]

Table S1

Table S1 The difference in translation of the proteins identified in the corresponding PSI-NDH supercomplex band between the *pbr1-1* mutant and wild-type or *PBR1*-overexpresson line OE-5 and wild-type

AGI Code	Gene Name	Ratio *pbr1-1*/WT	Unique Peptides	Coverage [%]	Ratio OE-5/WT	Unique Peptides	Coverage [%]
**Identified PSI-NDH subunit proteins encoded by nuclear genes**							
AT1G14150	PsbQ-F1	2.1176	7	34.2	1.2438	4	18.4
AT1G15980	NDF1	1.6427	17	35.1	1.2134	10	21.9
AT1G18730	NDF6	1.5555	5	21.7	2.5532	2	11.4
AT1G19150	Lhca6	0.3608	4	9.6	0.4359	1	3.7
AT1G30380	PsaK	0.3673	2	7.7	0.0398	2	7.7
AT1G31330	PsaF	1.921	8	35.7	1.2832	7	33
AT1G45474	Lhca5	3.4272	6	21.1	0.4138	4	18.4
AT1G52230	PsaH-2	0.4876	2	15.9	0.0488	3	15.2
AT1G55670	PsaG	5.164	8	21.9	2.2422	4	16.9
AT1G61520	Lhca3	6.2555	4	20.5			
AT1G64770	NDF2	0.5035	17	37.1	0.5424	12	27.3
AT1G74880	NdhO	0.1412	7	43.7	0.4279	2	12.7
AT2G39470	PPL2	3.7902	15	52.9	1.3057	8	26.5
AT3G01440	PsbQ-F2	0.3476	8	23.2	1.0504	6	20
AT3G47470	Lhca4	4.3476	2	7.2			
AT3G54890	Lhca1	3.3844	5	14.5	2.6166	5	13.3
AT3G61470	Lhca2	1.78	3	12.5			
AT4G09350	NdhT	3.1433	10	32.5	9.9423	3	14.9
AT4G12800	PsaL	16.488	4	17.4	8.5056	4	16.9
AT4G28750	PsaE-1	1.1664	4	41.3	0.1629	4	41.3
AT4G37925	NdhM	1.5921	6	24.4			
AT4G39710	FKBP16-2	2.2123	7	33.2	1.5079	5	19.8
AT5G13120	CYP20-2	2.7223	14	42.5	2.3783	5	18.1
AT5G21430	NdhU	1.242	13	55.5	1.1125	8	34.4
AT5G58260	NdhN	1.4588	6	31.6	0.5906	5	26.3
**Identified proteins encoded by chloroplast DNA**							
ATCG00020	PsbA	0.6125	7	19.8	0.7286	6	18.7
ATCG00120	AtpA	1.3368	7	17.6	0.0584	2	4.9
ATCG00130	AtpF	1.2696	4	24.5			
ATCG00170	RpoC2	1.4241	56	39.5			
ATCG00180	RpoC1	2.6362	25	35.3			

Table S1 (continued)

AGI Code	Gene Name	Ratio *pbr1-1*/WT	Unique Peptides	Coverage [%]	Ratio OE-5/WT	Unique Peptides	Coverage [%]
ATCG00190	RpoB	0.9711	29	25.7			
ATCG00270	PsbD	1.5595	5	20.4	1.0647	4	14.2
ATCG00280	PsbC	1.251	13	25.4	0.5578	8	15
ATCG00330	Rps14	2.9414	6	47			
ATCG00340	PsaB	4.9407	10	11.6	1.4964	8	11.2
ATCG00350	PsaA	2.0651	17	14.5	1.0629	13	14.9
ATCG00420	NdhJ	8.058	5	36.1	0.063	1	4.4
ATCG00430	NdhK	2.8802	6	23.1	0.5334	4	15.1
ATCG00480	AtpB	0.9158	11	27.7	0.4022	2	5.4
ATCG00490	RbcL	1.5489	10	19.8			
ATCG00500	AccD	1.2878	20	34.8	0.6084	2	4.5
ATCG00520	Ycf4	2.5691	2	9.2			
ATCG00540	PetA	0.5267	2	5.3			
ATCG00560	PsbL	4.1081	1	36.8	1.4518	1	36.8
ATCG00570	PsbF	2.4309	1	20.5	0.8159	1	20.5
ATCG00580	PsbE	2.1875	3	36.1	0.6369	3	36.1
ATCG00660	Rpl20	0.5377	9	33.3			
ATCG00680	PsbB	3.0059	18	26.6	1.7838	6	9.6
ATCG00710	PsbH	1.9102	3	37	0.6031	2	28.8
ATCG00740	RpoA	1.2885	11	27.1			
ATCG00770	Rps8	0.8584	4	26.1			
ATCG00790	Rpl16	0.2778	5	37.8			
ATCG00800	Rps3	1.2406	17	43.6			
ATCG00860	Ycf2	1.1399	27	12.6			
ATCG00890	NdhB	1.3112	3	6.4			
ATCG00900	Rps7	0.1823	8	40.6			
ATCG01010	NdhF	7.9428	11	12.7	0.9471	6	6.8
ATCG01050	NdhD	1.2344	4	9.4	1.6428	3	6.2
ATCG01090	NdhI	1.8408	6	33.7	1.8149	6	34.9
ATCG01100	NdhA	1.0884	3	5.6	1.9675	3	6.7
ATCG01110	NdhH	1.2405	14	26.2	0.9328	8	16.5
ATCG01130	Ycf1	0.3408	39	19.1	1.54	7	3.8

*For comparative analysis, the leaves of wild-type and *pbr1-1* or overexpression line of *PBR1* (OE-5) were pulse-labeled with 200 mg/L $^{13}C_6{}^{15}N_4$ L-Arginine (“heavy”, H) and $^{15}N_4$ L-Arginine (“medium heavy”, M), respectively. Gel slices corresponding to NDH-PSI supercomplex were excised and extracted peptides were analyzed by LC-MS/MS on a high performance mass spectrometer (LTQ-Orbitrap XL,Thermo Finnigan, San Jose, CA). The ratio of peak intensities of M versus H peptides reflects difference in translation of the corresponding proteins since the newly synthesized proteins incorporate either the M or H amino acids.
