# Supplementary material for: PBR1 selectively controls biogenesis of photosynthetic complexes by modulating translation of the large chloroplast gene Ycf1 in Arabidopsis
Source: Cell Discov. 2016 May 10;2:16003–. doi: 10.1038/celldisc.2016.3 (PMC4870678; doi:10.1038/celldisc.2016.3)
Supplement: Supplementary Table S2 [file celldisc20163-s13.pdf]

Table S2

Table S2 The primer sequences for constructions and gene expression analysis

Primer Name	Sequence (5'-3')
**Primers for constructions**	
GC-F	ACTGGATCCGTACACATTCTTGCCACGTGTTC
GC-R	GAGCTGCAGTCGATTGCTGATTTTGATTCCGTG
OE-F	TCAGTCGACATGCAAACGCTTCTCTGTCAG
OE-R	TATCCCGGGTCACTGACTCTCGAACTTGAAC
GUS-F	TCAAGCTTGTACACATTCTTGCCACGTGTTC
GUS-R	ATGGATCCCTTTTTTCCTCTTATCCGCCGCG
YFP-F	GGATCCATGCAAACGCTTCTCTG
YFP-R	CTCGAGCTGACTCTCGAACTTGAAC
RC-F	CTGGATCCATGCAAACGCTTCTCTGTCAG
RC-R	CTGAATTCTCACTGACTCTCGAACTTGAAC
Ycf-A-F	CACAGTCGACGCTAGCATGGTTTTTCAATCTTTTATAC
Ycf-A-R	CCCGGGTCTAGAAAGCTTCAATTCTATTTAAAAATTC
Ycf-B-F	TTGAAGCTTTAGATAAGGAATGG
Ycf-B-R	TTTTCTAGAAAAGAAGAGACTAAAC
Ycf-C-F	TTTTCTAGAAAAATATAATATTCGG
Ycf-C-R	CACACCCGGGTTAAGGAATTGGAAATCGCG
RbcS-TP-F	CACAGCTAGCTTCGGAATCGGTAAGGTCAG
RbcS-TP-R	CACAGTCGACATGGCTTCCTCTATGCTCTC
Ycf-5’UTR-F	TAATACGACTCACTATAGGGTATCCCTTTGGTGTCATTGAC
Ycf-5’UTR-R	GTCGACCACAAACCTCCCTTTTTTC
Ycf-3’UTR-F	TAATACGACTCACTATAGGGTCATAAATACTTTTTACTATA
Ycf-3’UTR-R	GTCGACATAATTGGTATCTATGTGATTG
PsaA-5’UTR-F	TAATACGACTCACTATAGGGAATTCTAAGTATCTATCATC
PsaA-5’UTR-R	GTCGACTGAGTCCTCCTCTTTCCGGAC
RbcL-5’UTR-F	TAATACGACTCACTATAGGGTGATTAGTTGATAATTTTGTG
RbcL-5’UTR-R	GTCGACAAGTCCCTCCCTACAAGTCA
Ycf1-CC1-Y2H-F	GAATTCGGATCCTGGATAAACAAGATTC
Ycf1-CC1-Y2H-R	CTGCAGATTTTTTATAAGCGTTTGATC
Ycf1-CC2-Y2H-F	GAATTCAAAAAAGATTCAATAATAAG
Ycf1-CC2-Y2H-R	CTGCAGAAAAAAAATGGATTTACGAA
PsaA-Y2H-F	CTGCAGATGATTATTCGTTCGCCGGAAC
PsaA-Y2H-R	CTCGAGTCCTACTGCAATAATTCTTGC
PsaB-Y2H-F	CTGCAGATGGCATTAAGATTTCCAAGG
PsaB-Y2H-R	AAGCTTACCGAATTTGCCCGATGTGG
PetA-Y2H-F	CTGCAGATGCAAACTAGAAATACCTTT
PetA-Y2H-R	AAGCTTAAAATTCATTTCGGATAATTG
AtpB-Y2H-F	CTGCAGATGAGAACAAATCCTACTAC
AtpB-Y2H-R	CTCGAGTTTCTTCAATTTACTCTCCA
Ycf4-Y2H-F	CTGCAGATGAGTTGGCGATCAGAATC
Ycf4-Y2H-R	CTCGAGAAATACTTCAATTGGTACAC

Table S2 (continued)

Primer Name	Sequence (5'-3')
**Primers for RT-PCR, qRT-PCR and northern blot analysis**	
ACTIN2-F	GCCATCCAAGCTGTTCTCTC
ACTIN2-R	GCTCGTAGTCAACAGCAACAA
PBR1-qRT-F	CTAAGAGAGATCTTGTCTGG
PBR1-qRT-R	AGAAGAATTGGATCGGTGAGC
Ycf1-qRT-F	GAAATGGAAGAAATCCGAGTG
Ycf1-qRT-R	CGAAAACGAGAGTTACAAATGG
Ycf1-Northern-F	TCAAATGAAAAAGAATCCCTTC
Ycf1-Northern-R	TCGCAATTGAAAACGCGAATATC
rbcL-Northern-F	AACCAAGGATACTGATATCTTGGC
rbcL-Northern-R	ATCGTCCTTTGTAACGATCAAGGC
psaA-Northern-F	TCCTAAAGAAATACCGCTTCC
psaA-Northern-R	ATCCACATGTGATGTGTGAAC
